# A vegan diet signature from a multi-omics study on different European populations is related to favorable metabolic outcomes

**DOI:** 10.1080/19490976.2025.2593050

**Published:** 2025-12-04

**Authors:** Anna Ouradova, Giulio Ferrero, Miriam Bratova, Nikola Daskova, Alena Bohdanecka, Klara Dohnalova, Marie Heczkova, Karel Chalupsky, Maria Kralova, Marek Kuzma, István Modos, Filip Tichanek, Lucie Najmanova, Barbara Pardini, Helena Pelantová, Sonia Tarallo, Petra Videnska, Jan Gojda, Alessio Naccarati, Monika Cahova

**Affiliations:** a Department of Internal Medicine, Kralovske Vinohrady University Hospital and Third Faculty of Medicine, Charles University, Prague, Czech Republic; b Department of Clinical and Biological Sciences, University of Turin, Turin, Italy; c Department of Computer Science, University of Turin, Turin, Italy; d Institute for Clinical and Experimental Medicine, Prague, Czech Republic; e First Faculty of Medicine, Charles University, Prague, Czech Republic; f Czech Centre for Phenogenomics, Institute of Molecular Genetics of the Czech Academy of Sciences, Prague, Czech Republic; g Ambis University, Department of Economics and Management, Prague, Czech Republic; h Department of Informatics, Brno University of Technology, Brno, Czech Republic; i Institute of Microbiology of the Czech Academy of Sciences, Prague, Czech Republic; j Italian Institute for Genomic Medicine (IIGM), c/o IRCCS Candiolo, Turin, Italy; k Mendel University, Department of Chemistry and Biochemistry, Brno, Czech Republic

**Keywords:** Vegan diet, shotgun metagenomic sequencing, gut microbiota, untargeted serum metabolomics, serum lipidomics

## Abstract

Vegan and omnivorous diets differ markedly in composition, but their effects on the gut microbiome, metabolome, and lipidome across populations remain insufficiently characterized. While both diet and country of origin influence these molecular layers, the relative contribution of diet versus country-specific factors has not yet been systematically evaluated within a multi-omics framework.

In this cross-sectional, bicentric, observational study, we profiled healthy vegans (*n* = 100) and omnivores (*n* = 73) from the Czech Republic and Italy using integrated microbiome, metabolome, and lipidome analyses. Findings were subsequently validated in an independent cohort (*n* = 142).

Significant differences across all omics layers were observed for both country and diet. The predictive models confirmed diet-associated separation, with validation cohort AUCs of 0.99 (lipidome), 0.89 (metabolome), and 0.87 (microbiome). Functional metagenome analysis revealed enrichment of amino acid biosynthesis, inositol degradation, and the pentose phosphate pathway in vegans, while omnivores presented greater potential for amino acid fermentation, fatty acid biosynthesis, and propanoate metabolism. Linear models identified a robust, country-independent “vegan signature” consisting of 27 lipid metabolites, five non-lipid metabolites, and 11 bacterial species. Several lipid features associated with an omnivorous diet were inversely related to the duration of vegan diet adherence. Some of the vegan-associated metabolites and bacteria have been previously linked to favorable cardiometabolic profiles, although causality remains to be established.

These findings demonstrate that vegan diets are associated with reproducible, country-independent molecular and microbial signatures. Our results highlight diet-driven shifts in host–microbiota interactions and provide a framework for understanding how dietary patterns relate to host–microbiota interactions.

## Introduction

While a vegan diet carries certain nutritional risks, it is also associated with a reduced risk of cardiometabolic disease.^1-3^ Whether this benefit arises directly from dietary composition or through gut microbiota-mediated mechanisms remains unsolved. We have recently shown that the health effects of a vegan diet may be conveyed by the metabolic performance of the gut microbiota.^4^ However, it remains unclear whether these effects extend across different geographical regions and dietary traditions, and whether additional nutritional drivers beyond fiber are involved. Long-term vegans living in countries with distinct cultural and dietary backgrounds may provide a suitable model population to explore these relationships further.

Most previous studies investigating the impact of vegan versus omnivorous diets have focused on the gut microbiome composition. The extent to which diet influences microbial communities strongly depends on the methodological approach: less sensitive methods (e.g., culture-based approaches, denaturing gradient gel electrophoresis, or short 16S amplicon sequencing) have generally reported only modest differences,^5-8^ whereas whole-metagenome shotgun sequencing (WMGS) has revealed marked taxonomic shifts between vegans and omnivores at lower taxonomic levels.^9^^,^^10^ However, even WMGS studies have concentrated almost exclusively on the microbiome, while studies addressing the impact of long-term vegan diets on the host metabolome remain rare, typically limited to small cohorts, and have yielded inconsistent findings.^5^^,^^8^^,^^11^

Although it is well established that both microbiome composition and its functional outputs are strongly influenced by geographic and environmental factors, it remains unclear how these factors interact with dietary patterns, particularly long-term adherence to a vegan diet. Previous large-scale, cross-country cohort studies have demonstrated distinct associations between dietary aspects and the gut microbiome structure.^10^ However, the impact of a vegan diet on the host metabolome and lipidome has not been systematically addressed. To date, no cross-country multi-cohort study has integrated gut microbiome profiles with other biochemical layers such as the serum metabolome and lipidome, to disentangle the specific contribution of diet from the influence of geography.

In the present study, we aimed to fill this gap by integrating multiple omics approaches – metagenomics, serum metabolomics, and serum lipidomics – to analyze and independently validate the gut microbiome and serum MEtabolome (MIME) composition in vegans and omnivores from the Czech Republic and Italy. Specifically, we sought to determine whether: (i) MIME composition differs between vegans and omnivores according to the country of origin; (ii) diet-dependent but country-independent features characterize vegans; and (iii) any diet-dependent features may account for the observed health benefits of a vegan diet.

## Materials and methods

### Study design and study populations

**Figure 1.**
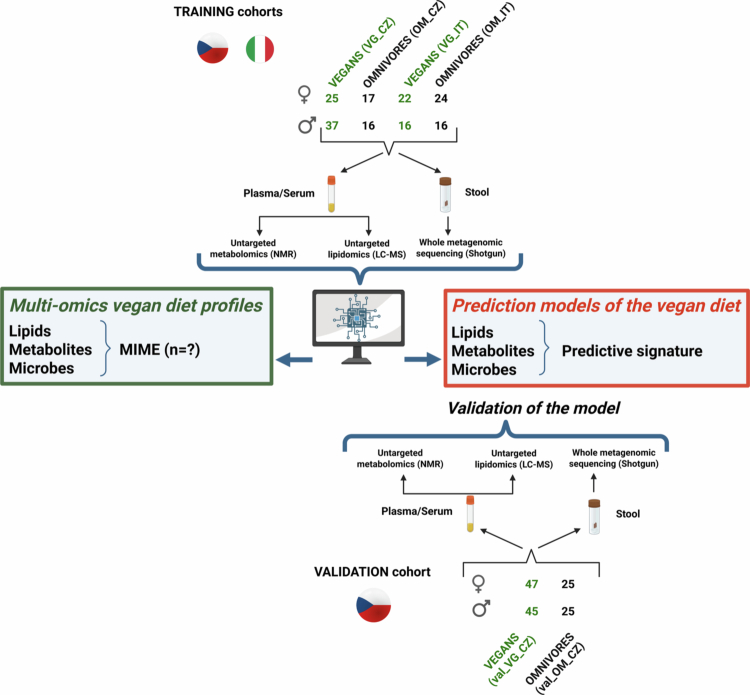
Design of the study: from the collection of samples in each study cohorts (training and validation) to the wet-lab and statistical analyses performed. The study cohorts are described in black (omnivores) and green (vegans); CZ indicates Czech and IT for Italian origin; and the number of participants according to sex is described using symbols ♂ for male and ♀ for female probands. The type of samples and methods for their evaluation are depicted, as well as further bioinformatic approaches employing the obtained results.

We performed a cross-sectional, observational study exploring the specific signature of a vegan diet across the microbiome, metabolome, and lipidome profiles in 3 European cohorts (two from the Czech Republic and one from Italy) with a training and test cohort approach. The training cohort consisted of 100 healthy vegan (VG) and 73 omnivore (OM) participants from the Czech Republic and Italy. Subjects were enrolled and screened between May 2017 and October 2019 for a cross-sectional comparison in both the Czech Republic (training CZ cohort, Prague)^5^ and Italy (IT cohort, Torino).^12^ The results were validated on an independent cohort of 142 Czech subjects (92 vegans and 50 omnivores) recruited in the Czech Republic (validation CZ cohort, Prague) between September 2021 and June 2022. In accordance with the inclusion criteria, vegans self-reported the strict exclusion of all animal products (≥1 y), and the omnivore group included subjects without any dietary restrictions who consumed meat and other animal products on a daily basis. In all the groups, the exclusion criteria were age under 18 y, obesity, chronic diseases related to metabolism, diseases of the digestive tract, antibiotic therapy in the past three months, pregnancy, and any chronic medication (excluding hormonal contraception). Each subject underwent a basic medical check-up with an anthropometric examination (height, weight, BMI, waist circumference, and waist-to-hip ratio); body composition (only for training and validation CZ cohorts) was determined by bioimpedance analysis (Nutriguard-M). The analytical pipeline is depicted in Figure 1. All participants provided written informed consent following the Declaration of Helsinki before participating in the study. The research protocol was approved by the Institutional Review Board of University Hospital Kralovske Vinohrady (training CZ cohort: EK-VP/26/0/2017; validation CZ cohort: EK-VP/39/0/2020) and by the Ethics Committee of Azienda Ospedaliera SS. Antonio e Biagio e Cesare Arrigo di Alessandria, Italy (IT cohort: Colorectal miRNA_CEC2014 and subsequent amendments).

### Dietary intake assessment

Dietary records and stool samples were obtained no longer than a week after the clinical visit. In the CZ cohorts, a three-day prospective food record supervised by a trained dietitian was used to assess the macronutrient composition and fiber content of the diet.^5^ Two working and one weekend days were recorded. The volunteers were educated, and instructions were given for portion size estimation and recording of foods in sufficient detail to obtain an accurate estimate of consumed portions. A portion estimation guide was used as a reference. The daily intake of carbohydrates, lipids, proteins, and dietary fiber was calculated using the Nutriservis PROFI software (Forsapi Ltd) using the food database in this nutritional software. The energy and nutrient content of novel food products (such as vegan meat alternatives and ready-to-eat meals or snacks) were estimated by recipe simulation using labeled ingredients and nutrient content. The Nutriservis PROFI is continuously updated by adding those products or supplements recorded by dietitians. In cases where some foods from the dietary record were missing from the Nutriservis PROFI database, a dietitian manually entered them into the database. The daily intake of carbohydrates, lipids, proteins, and dietary fiber was calculated separately.

For the IT cohort, all the recruited subjects completed a validated self-administered Food Frequency Questionnaire (FFQ) assessing their usual diet, along with lifestyle and personal history data,^12^in accordance with the European Prospective Investigation into Cancer and Nutrition EPIC study standards.^13^ The FFQ consisted of 248 questions concerning 188 different food items and included pictures with two or three sample dishes of definite sizes or references to standard portion sizes. The nutrient compositions of individual food items were obtained from Italian food composition tables,^14^ and the average daily intake of macronutrients and micronutrients for each volunteer was estimated.

### Sample collection

Stool samples. For the CZ cohorts, stool was collected at home and immediately stored at –20 °C in the native state until it was transported frozen to the laboratory. Once thawed, a four-fold volume of water was added to the sample (up to 10g), which was subsequently homogenized using stomacher (BioPro). Immediately after homogenization, an aliquot (600 µl) was taken for DNA analysis. For the IT cohort, stool samples were collected at home into nucleic acid collection tubes and transport tubes with RNA/DNA stabilizing solution (Norgen Biotek Corp.) and kept at room temperature until delivery to the laboratory, where they were stored at –80 °C until DNA extraction.

Blood and serum samples. For all the subjects, blood samples were drawn from the median cubital vein into a Vacutainer tube. For serum, the blood was left standing on the bench for 30 min to clot and then separated by centrifugation. For the plasma samples, whole blood was collected into a Vacutainer with an anticoagulant, immediately mixed by gently inverting the tube five times and then separated by centrifugation.

### DNA extraction and gut microbiome analysis

Bacterial DNA was isolated from the stool samples using the QIAamp PowerFecal Pro DNA Kit (Qiagen) and the composition of the microbiome was determined by whole metagenome shotgun sequencing as described in Thomas et al.^15^ Briefly, the library was prepared using the Illumina DNA Prep (M) Tagmentation Kit (Illumina) according to the manufacturer’s instructions with normalized 24 ng of DNA input in 30 µl of nuclease-free water. More details on library preparation are reported in the Supplementary materials. The purified libraries were quantified using a fluorometric-based method (Qubit) and the library quality was checked via the Agilent Fragment Analyser 5200 or 2100 Bioanalyser (Agilent) using the High Sensitivity DNA kits. All the libraries were pooled to a total molarity of 150 nM. The final library pools were sequenced on an Illumina NovaSeq6000 sequencer. The read preprocessing (adapter trimming and removal of low-quality reads), the alignment on PhiX control, and the human genome (hg38) were performed as described in the study by Thomas et al.^15^, using the pipeline available at https://github.com/SegataLab/preprocessing reads number, length, and base qualities were extracted using Seqkit v2.10.1. Taxonomic profiling was performed with MetaPhlAn 4.1^15^^,^^16^ and the ChocoPhlAn vJun23 database. MetaPhlAn was applied in the default setting and with a stat_q value equal to 0.1. Microbial pathway abundances were estimated using HUMAnN 3.9 with default settings and using the full UniRef90 as a reference database.

For pathway analyses, we used unstratified community-level abundances from HUMAnN (rows without a taxonomic suffix), which represented the total pathway signal in the community. Across cohorts, unmapped reads accounted for approximately 20%–23% of the data, unintegrated reads for 72%–74%, and among the classified pathways, approximately 9%–11% of the signals could not be taxonomically assigned.

### Lipidomics analysis

Lipids were extracted via phase separation using methanol/methyl tert-butyl ether as described previously.^17^ Briefly, a 6564 Dual AJS ESI Q-TOF mass spectrometer (MS) coupled with a 1290 Infinity II LC System (Agilent) or an Orbitrap ID-X Tribrid coupled with a Vanquish Horizon UHPLC system (both Thermo Fisher Scientific) was used to analyze the extracted serum samples reconstituted in methanol/2-propanol (1/1, v/v). Chromatographic separation was performed on an Acquity UPLC BEH C18 column (130 Å, 1.7 µm, 2.1 mm × 150 mm) (Waters) at 55 °C using a gradient of ACN/water (A, 60/40, v/v, with 10 mM ammonium formate and 0.1% formic acid) and 2-propanol/ACN (B, 90/10, v/v, with 10 mM ammonium formate and 0.1% formic acid). The tuning mix (Agilent, Santa Clara, USA) was used for instrument calibration. Peaks from MS measurement were assigned using mass and RT information from a lipid library created based on iterative MS/MS measurements. The data were processed in MassHunter Lipid Annotator 1.0.54.0 and MassHunter Quantitative Analysis 10.1.733.0 (Agilent), or in MZmine 3.9.0.^18^ LCMS-grade solvents and modifiers were purchased from Sigma–Aldrich: methanol, water, methyl tert-butyl ether, 2-propanol, acetonitrile, ammonium formate and formic acid. Splash Lipidomix Mass Spec Standard containing, among others, standards of phosphatidylcholine (PC) 15:0_18:1(d7), phosphatidylethanolamine (PE) 15:0_18:1(d7), phosphatidylserine (PS) 15:0_18:1(d7), lysophosphatidylcholine (LPC) 18:1(d7), lysophosphatidylethanolamine (LPE) 18:1(d7), cholesterol ester (CE) 18:1(d7), diglyceride (DG) 15:0_18:1(d7), triglyceride (TG) 15:0_18:1(d7)-15:0 and sphingomyelin (SM) 18:1(d9) were acquired from Avanti Polar Lipids. PC 22:1_22:1 was purchased from Avanti Polar Lipids.

### NMR analyses

Serum samples were analyzed after protein precipitation as described previously.^19^ A 220  µl serum aliquot was mixed with 440  µl cold methanol. The mixture was kept in a freezer at –20 °C for 30 min and then centrifuged at 18,620 g for 10 min at 4 °C. The supernatant was transferred into a fresh vial and vacuum dried. The dry supernatant was dissolved in 450  µl D_2_O with 50  µl 1.5 M phosphate buffer and 50 μl 0.1% TSP and then transferred into a 5 mm NMR tube.

NMR data were acquired on a 600  MHz Bruker Avance III spectrometer (Bruker BioSpin) equipped with a 5 mm TCI cryogenic probe head. All the experiments were performed using Topspin 3.5 software at 300 K with automatic tuning and matching, shimming and adjusting the 90° pulse length for each sample. Serum data were analyzed from Carr–Purcell–Meiboom–Gill (CPMG) spectra acquired by cpmgpr1d pulse sequence with the following acquisition parameters: number of scans (NS) = 192, spectral width (SW) = 20 ppm, 64k data points (TD), relaxation delay for water presaturation d1 = 4 s, echo time 0.3  ms, and loop for T2 filter 126. A *J*-resolved experiment (NS = 2, SW = 16, TD = 8k, number of increments = 40, SW = 78.125 Hz in the indirect dimension, d1 = 2 s) was performed on each sample to facilitate metabolite identification. Additional heteronuclear single quantum correlation (HSQC) and total correlation spectroscopy (TOCSY) experiments were executed for selected samples.

The acquired data were processed with Topspin 3.5 software. The CPMG spectra were line broadened (0.3 Hz), automatically phased, baseline corrected, and referenced to the signal of the TSP. The regions with signals from water and methanol were excluded, and then the spectra were normalized using probabilistic quotient normalization (PQN) method^20^ to the pooled lean healthy group. Individual metabolites were identified using Chenomx software (Chenomx Inc.) and their proton and carbon data were then compared to those of the HMDB database.^21^ Metabolite concentrations were expressed as normalized intensities of corresponding signals in CPMG spectra. The list of metabolites analyzed and a representative ^1^H NMR spectrum of serum with quantified metabolites are reported in the **S**upplementary materials.

### Laboratory analyses

Parameters of glucose homeostasis (fasting plasma glucose, glycated hemoglobin (HBA1c), C-peptide, OGTT glucose, and insulin levels) and lipid profile (total cholesterol, high-density lipoprotein cholesterol, low-density lipoprotein cholesterol and triglycerides) were assessed in a certified hospital laboratory using commercially available kits.

### Data analysis

All the analyses were conducted in R v4.4.1 (R Core Team, 2023). Visualizations were generated with ggplot2 v3.5.1. For comparisons of health- and metabolism-related variables, data normality was assessed using the Shapiro–Wilk test, group differences with the Kruskal–Wallis test followed by Dunn’s post-hoc test, and categorical variables with Fisher’s exact test.

For microbiome analyses, both taxonomic profiles (species-level genomic bins, SGBs) and functional pathways were treated as compositional data. Zeros were imputed using log-ratio singular value decomposition (zCompositions v1.5.0−4). For alpha diversity, all detected SGBs were included without prevalence-based filtering. Richness was defined as the number of SGBs with relative abundance >0, while Shannon and inverse Simpson indices were computed using the vegan v2.6−6.1 package. The alpha diversity indices across diet groups were compared with the Wilcoxon rank-sum test. For all other microbiome analyses, low-abundance taxa were filtered out with *nearZeroVar* (caret v7.0−1). This function identifies taxa associated with a single unique value or with few unique values relative to the number of samples (*uniqueCut* = 10 by default) and with a large ratio between the frequency of the most common value and that of the second most common value (*freqCut* = 95/5 by default). Moreover, only features with prevalence >30% were retained for linear models and predictive modeling.

Beta diversity was analyzed using Bray–Curtis dissimilarities (*vegdist*, vegan). Principal component analysis (*prcomp*, base R), non-metric multidimensional scaling (*metaMDS*, vegan), and permutational multivariate analysis of variance (*adonis2*, vegan) were applied. Robustness was examined using the 20 additional distance metrics available in *vegdist* (vegan).

Diet-associated features across omics layers (microbiome, functional pathways, metabolome, lipidome) were identified with linear models fitted in the training cohorts. The models included *diet* (vegan vs. omnivore), *country* (Italy vs. Czech Republic), and their interaction (*diet × country*) as fixed-effect predictors. Omics variables were used as outcomes and transformed using log2 (metabolome, lipidome) or clr transformation (microbiome, pathways). From each model, we extracted both the average diet effect across the two countries and the country-specific diet effects, using the emmeans package v1.10.4. Features with significant average diet effects (FDR<0.05) were validated in an independent Czech cohort, and the results were visualized with forest plots. To evaluate whether omics features that differ between vegans and omnivores also vary within the vegan group itself, depending on the duration of the vegan diet, additional models included vegan *diet duration*, *country*, *diet duration × country* interaction, and *age* as fixed-effect predictors (age was included owing to its correlation with diet duration).

Predictive models were fitted using elastic net logistic regression (glmnet v4.1−8) separately for each omics dataset. These models are not intended for practical prediction but rather for quantifying the strength of the signal separating dietary groups and providing a complementary perspective on which features are most strongly associated with diet. Because of the expected high collinearity among features, we deliberately chose low *α* values (0, 0.2, 0.4), making the models closer to ridge regression and thus not performing feature selection in the strict sense. The features were standardized (zero mean, scaled by two SD using arm v1.12−2). For each *α*, 10-fold cross-validation was used to determine the *λ* corresponding to the most regularized model within one SE of the minimum deviance (*lambda.1se*). To quantify uncertainty, the entire procedure was repeated on 500 bootstrap resamples of the training data; in each iteration, the model was trained on resampled data and evaluated on out-of-bag samples. Performance was summarized as the mean out-of-sample AUC (pROC v1.18.0) with 95% confidence intervals (2.5th and 97.5th percentiles of the out-of-sample AUC distribution across bootstrap resamples), both separately for each country and for the combined training cohorts. Final models trained on the combined training cohorts were externally validated in the independent Czech cohort.

Correlation networks were built from significant inter-omics Spearman correlations (adjusted *p* < 0.05, where the direction of correlation was consistent across all three cohorts) and visualized in Cytoscape v3.10.3.

A strain-level analysis was performed on metagenomic data using StrainPhlAn v4.0 and focused only on the diet-associated SGBs associated with a defined reference in NCBI. Details on the parameters used are given in Supplementary materials.

Effect sizes and standard errors for four additional cohorts reported in^10^ (DeFilippisF_2019, P1, P3 UK22A, P3 US22A) and for the meta-analysis of Fackelmann et al. study,^10^ were obtained from Supplementary Table 11 of the publication. Taxon-specific health scores were obtained from the ZOE Microbiome Health Ranking 2024 (https://zoe.com/microbiome-ranking). The mean ranks of taxa enriched in vegans or omnivores were compared using the Mann‒Whitney U test.

All code and full model specifications are available at: https://filip-tichanek.github.io/ItCzVegans/.

## Results

### Description of the groups and clinical outcomes

The characteristics of the Italian and Czech vegans (VG_IT; VG_CZ) and omnivores (OM_IT; OM_CZ) in the training cohort are shown in Table 1 and Supplementary Table 1. The dietary records confirmed higher fiber intake and a lower intake of saturated fatty acids in the VG group than in the respective OM groups. Both IT dietary groups had a higher intake of monounsaturated fatty acids than the Czech groups. VG_IT had a lower intake of all the assessed nutrients when compared with the VG_CZ, which might have resulted from the different methodologies used to collect dietary data (3-d prospective record in the training CZ cohort vs. FFQ in the IT cohort). Though both vegan and omnivore participants were healthy overall, significant differences in clinical outcomes were observed. The total cholesterol (TC) and LDL-cholesterol (LDL-c) concentrations were lower in the VG_CZ (TC: *Z* = 5.34, *p *< 0.001; LDL-c: *Z* = 4.34, *p *< 0.001) and VG_IT groups (TC: *Z* = 3.59, *p* = 0.001; LDL-c: *Z* = 3.93, *p* = 0.001) than in the respective OM groups. Compared with the OM_CZ group, the VG_CZ group also presented lower HDL cholesterol (HDL-c) (*Z* = 3.61, *p* = 0.002) and lower total cholesterol (TC) levels than the VG_IT group did (*Z* = 3.03, *p* = 0.004). The serum triglyceride (TAG) concentration was higher in both IT groups, independent of the diet (OM: *Z* = –4.25, *p* = *p *< 0.001; VG: *Z* = –4.54, *p* < 0.001). The characteristics of the validation cohort showed similar patterns, as shown in Supplementary Table 2. The results from additional clinical analyses were available only for the CZ cohort. Among them, several parameters of glucose metabolism were different: lower HbA1c concentration (*W* = 1354, *p* = 0.009) and a trend toward lower 120 min glycemia during the oral glucose tolerance test (*W* = 1232, *p* = 0.075). In addition, a lower serum zonulin concentration (*W* = 1301, *p* = 0.039) was detected in the VG (Table 1).

**Table 1.** Group characteristics of vegans and omnivores from the Czech (CZ) and Italian (IT) training cohorts.

	VG_CZ	OM_CZ	VG_IT	OM_IT	Dunn’s multiple comparison test			
					VG_CZ vs OM_CZ	VG_IT vs OM_IT	OM_CZ vs OM_IT	VG_CZ vs VG_IT
**General characteristics**								
Sex [F/M]	25/35	17/16	22/16	24/16	0.088	0.999	0.624	0.017
Age [years]	30.9 (10.5)	31.3 (11.0)	37.8 (19.6)	39.3 (19.2)	0.745	0.754	0.019	0.008
BMI [kg.m^−2^]	21.6 (3.6)	22.7 (4.3)	21.1 (3.8)	23.0 (5.3)	0.300	0.130	0.700	0.220
Waist circumference [cm]	76.0 (9.0)	78.0 (16.0)	78.0 (8.0)	81.0 (17.3)	0.990	0.263	0.358	0.856
**Lipid metabolism**								
TC [mmol.L^−1^]	3.3 (0.8)	4.3 (1.1)	3.9 (0.9)	4.5 (1.5)	<0.001	0.001	0.263	0.001
HDL-C [mmol.L^−1^]	1.4 (0.4)	1.7 (0.7)	1.4 (0.4)	1.4 (0.6)	0.002	0.653	0.218	0.189
LDL-C [mmol.L^−1^]	1.7 (1.5)	2.4 (1.2)	1.8 (0.5)	2.4 (1.0)	<0.001	<0.001	0.378	0.073
Triacylglycerols [mmol.L^−1^]	0.7 (0.4)	0.7 (0.5)	0.9 (0.6)	1.2 (1.1)	0.309	0.148	<0.001	<0.001
**Glucose metabolism**								
Fasting glucose [mmol.L^−1^]	4.7 (0.4)	4.8 (0.3)	N/A	N/A	0.140	N/A	N/A	N/A
2 h OGTT glucose [mmol.L^−1^]	5.5 (1.3)	5.9 (1.4)	N/A	N/A	0.075	N/A	N/A	N/A
OGTT AUC_glucose_ [mmol. L^−1^ x 120 min^−1^]	184 (159)	255 (137)	N/A	N/A	0.190	N/A	N/A	N/A
OGTT AUC_insulin_ [mIU. L^−1^ x 120 min^−1^]	3143 (2603)	4416 (1938)	N/A	N/A	0.004	N/A	N/A	N/A
Insulin [mIU.L^−1^]	3.4 (1.7)	3.9 (2.7)	N/A	N/A	0.460	N/A	N/A	N/A
C-peptide [pmol. L^−1^]	229 (79)	232 (103)	N/A	N/A	0.310	N/A	N/A	N/A
HbA1c [mmol.mol^−1^]	30.0 (4.0)	32.0 (2.5)	N/A	N/A	0.009	N/A	N/A	N/A
**Iron metabolism**								
Ferritin [ng.mL^−1^]	30.8 (37.2)	49.1 (73.2)	25.0 (41.7)	50.0 (64.2)	0.085			
Transferrin [g.L^−1^]	2.5 (0.8)	2.5 (0.5)	2.3 (0.6)	2.4 0.3)	0.730	0.760	0.049	0.049
**Inflammation**								
CRP [mg.L^−1^]	0.045 (0.028)	0.074 (0.081)	N/A	N/A	0.310	N/A	N/A	N/A
**Intestinal permeability**								
Zonulin [ng.ml^−1^]	25.5 (33.9)	18.1 (27.3)	N/A	N/A	0.039	N/A	N/A	N/A
**Stool characteristics**								
pH in feces	7.3 (0.7)	6.9 (0.8)	N/A	N/A	0.005	N/A	N/A	N/A
dry mass (%)	25.1 (9.9)	20.3 (8.8)	N/A	N/A	0.002	N/A	N/A	N/A

Data are presented as the median (interquartile range). The overall differences among groups were evaluated using Kruskal-Wallis test followed by the Mann‒Whitney U test. BMI, body mass index; CRP, C-reactive protein; HDL-C, high density lipoprotein–cholesterol; LDL-C, low density lipoprotein–cholesterol; N/A not applicable; n.s., not significant TC, total cholesterol.

### Multi-omics characterization of the investigated groups

Given the marked country-specific differences between Czech and Italian diets, we conducted an exploratory analysis to assess whether dietary choice exerts a stronger influence than the country of origin when evaluated using individual omics datasets.

**Figure 2.**
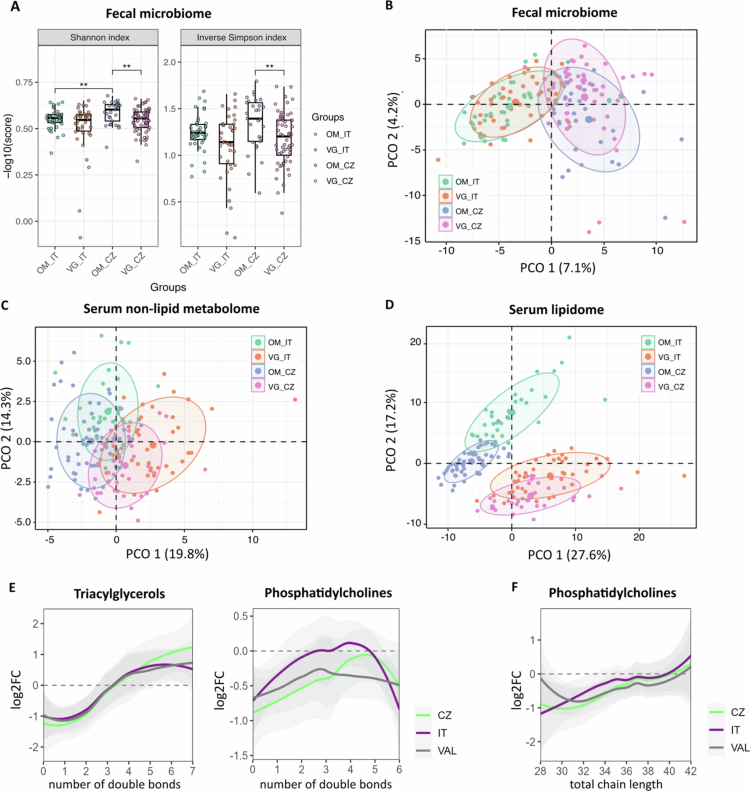
Multi-omics characterization of the investigated groups. (A) Boxplot representing the microbial alpha diversity measures across the study groups. *P* value from the Wilcoxon rank-sum test. **p < 0.01. (B-D) PCA score plots relative to (B) the gut microbiome, (C) the serum non-lipid metabolome, and (D) the serum lipidome. The explained variance of each component is included in the axis labels. The large points represent the centroids of each group. (E) Saturation of fatty acyl moieties in triacylglycerols (left) and in phosphatidylcholines (right). F) Length of the fatty acyl moieties in phosphatidylcholines. The curves represent moving regression (LOESS) fitted to individual values of log_2_ fold change between vegans and omnivores. A 95% confidence interval is shown as a light gray area.

Shotgun metagenomic sequencing generated a median of 34.18 million human-unmapped reads that were used for the taxonomic profiling (Supplementary Table 3A). Analysis at the SGB level revealed 1,724 and 1,636 taxa identified in the gut microbiome of the IT and CZ cohorts, respectively. At this level, 329 SGBs were uniquely found in the Italian gut microbiome, while 417 SGBs were unique to Czech subjects (Supplementary Table 3B), all belonging to low-abundance and rare taxa. The *α* diversity indexes, calculated on the full SGB dataset, are shown in Figure 2A. According to the Shannon index, the Czech omnivore population tended to have a more diverse microbiome than the Italian population did, but the difference did not reach statistical significance (median = 3.8 and 3.4, respectively, W = 791, *p* = 0.098). Furthermore, VG had a less diverse microbiota than OM in the training CZ cohort (median = 3.5, W = 1172, *p* = 0.005) but not in the Italian cohort (median = 3.4, *W* = 875, *p* = 0.254). Similar conclusions could be drawn from the inverse Simpson index; however, for this parameter, the difference between the OM groups was not statistically significant (*W* = 739, *p* = 0.054). After the removal of rare and low abundant species (nearZeroVar filtering) and those detected in less than 30% of the analyzed samples (Supplementary Figure 1 and Supplementary Table 3B), further analyses were performed on the 299 shared taxa found in both groups, mostly assigned at the species level (Supplementary Table 3C). Beta diversity analysis showed a clear separation between the cohorts and dietary groups (PERMANOVA with Bray‒Curtis dissimilarity, *F* = 16.98, *p* = 0.001 and *F* = 2.27, *p* = 0.003, respectively; Supplementary Table 4A), and the results were consistent with those of the other 20 beta diversity distances, including the Jaccard index and Kulczynski (Supplementary Table 4A).

In the lipidome and non-lipid metabolome datasets, no compounds had to be removed, as all of them were detected in more than 30% of the samples. Overall, the PCA of the data showed that the microbiome, as well as the non-lipid (Supplementary Table 3D) and lipid (Supplementary Table 3E) metabolome compositions, differed significantly according to both the diet and the country (Figure 2B–D). Pairwise PERMANOVA unraveled statistically significant differences between all groups (Supplementary Table 4B). The lipidomic profiles of both the Italian and Czech VG followed a similar pattern in terms of lipid characteristics. Compared to OM, VG had lower saturated and oligo-unsaturated triglyceride levels and higher highly unsaturated triglyceride and phosphatidylcholine levels (Figure 2E). In addition, VG had lower levels of shorter phosphatidylcholines than OM (Figure 2F).

### Evaluation of diet-associated microbial and metabolic signatures

To evaluate how diet shapes host and microbial profiles independently of the country of origin, we implemented elastic net logistic regression models using microbial, lipid, and non-lipid metabolite features. The predictive models were built from data obtained from the training cohort, and their performance was evaluated using an out-of-bag bootstrap (internal validation) on the merged dataset or on the training CZ and IT cohorts separately. The models were then tested in an independent validation CZ cohort (external validation). The performances of the elastic-net regression models are shown in Table 2 and Supplementary Figure 2A–D, while the regression coefficients are provided in Supplementary Figures 3–6. The lipidome proved to be superior to all other data sources for subject classification, reaching AUCs of 0.99 (0.99; 1.00) and 0.93 (0.88; 0.98) in the internal and external validation, respectively. Based on metabolome data, the model reached an AUC of 0.95 (0.90; 0.99) in the internal validation cohort and 0.91 (0.86; 0.97) in the external validation. The discriminatory power of the model based on microbiome data was the least accurate, even though it still reached an internal AUC of 0.89 (0.81; 0.95) and an external AUC of 0.88 (0.81; 0.95). To control for country-specific bias, we also evaluated model performance separately in the Czech and Italian training cohorts. The results were identical for non-lipid and metabolome datasets, while the microbiome model performed better in the Czech cohort (AUC 0.94; 95% CI 0.85, 1.00) than in the Italian cohort (AUC 0.81; 95% CI 0.66, 0.92). Together, these results indicate that, despite differences between the two European cohorts, vegans and omnivores display reproducible, country-independent signatures across the lipidome, metabolome, and microbiome layers, underscoring the consistent influence of diet on host and microbial profiles.

**Table 2.** The discriminatory power of microbiome, lipidome, and metabolome features to identify dietary habits.

		Microbiome		Non-lipid metabolome		Lipidome	
		AUC	95% CI	AUC	95% CI	AUC	95% CI
Training	Training set	1.000	0.999; 1.000	0.988	0.970; 1.000	1.000	1.000; 1.000
	Out-of-sample (all)	0.887	0.805; 0.952	0.949	0.900; 0.989	0.999	0.995; 1.000
	Out-of-sample (CZ)	0.943	0.851; 1.000	0.950	0.883; 1.000	1.000	0.995; 1.000
	Out-of-sample (IT)	0.806	0.661; 0.924	0.950	0.858; 1.000	0.998	0.985; 1.000
V	**independent (CZ)**	**0.879**	**0.806; 0.951**	**0.912**	**0.859; 0.966**	**0.993**	**0.986; 1.000**

The model was developed separately for each omic considering the combined training dataset (CZ and IT cohorts), with the optimized alpha (mixing parameter) and lambda (penalty strength) values selected via 10-fold cross-validation. The alpha and lambda exact values were as follows: microbiome *α *= 0.4, *λ *= 0.028; non-lipid metabolome *α *= 0, *λ *= 0.027; and lipidome *α *= 0.2, *λ *= 0.004. Internal validation employs 500 out‑of‑bag bootstrap resamples: the out‑of‑sample AUC is the mean across resamples, and its 95% confidence interval (CI) is given by the 2.5th and 97.5th percentiles of the bootstrap distribution. The training‑set AUC and its CI were computed analogously from the in‑bag predictions. External validation was carried out on an independent Czech cohort; the reported AUC is the point estimate for that cohort, and its 95% CI was obtained with DeLong’s method. V, validation cohort.

### Identification of multi-omics variables characteristic of the vegan diet

**Figure 3.**
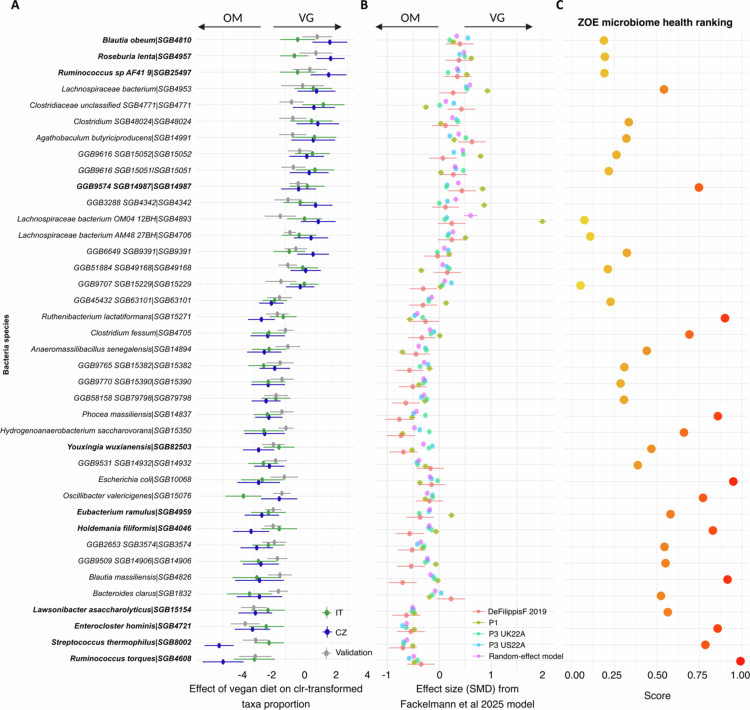
(A) Effects of a vegan diet on the levels of selected SGBs. The plot displays the estimated diet effects and 95% confidence intervals across all three cohorts (training CZ and IT cohorts and external validation CZ cohort), reporting only the variables that significantly differed between diets in the training cohorts. Positive estimated values/intervals of contrast indicate higher values of individual variables for the vegan group (than for the omnivore group). Variables marked in bold belong to the *vegan signature.* (B) Standardized mean difference (SMD) and standard error of the relative abundances of the SGB between omnivore and vegan subjects in a model adjusted for sex, age, and BMI for four cohorts from Fackelmann et al. (indicated as DeFilippisF_2019 and the PREDICT cohorts (P1, P3 UK22A, P3 US22A)) and the random-effect model estimated from the meta-analysis performed in the study. (C) Dot plot reporting, for each SGB, the ZOE microbial health rank. High ZOE rank values correspond to high positive associations with cardiometabolic risk factors.

**Figure 4.**
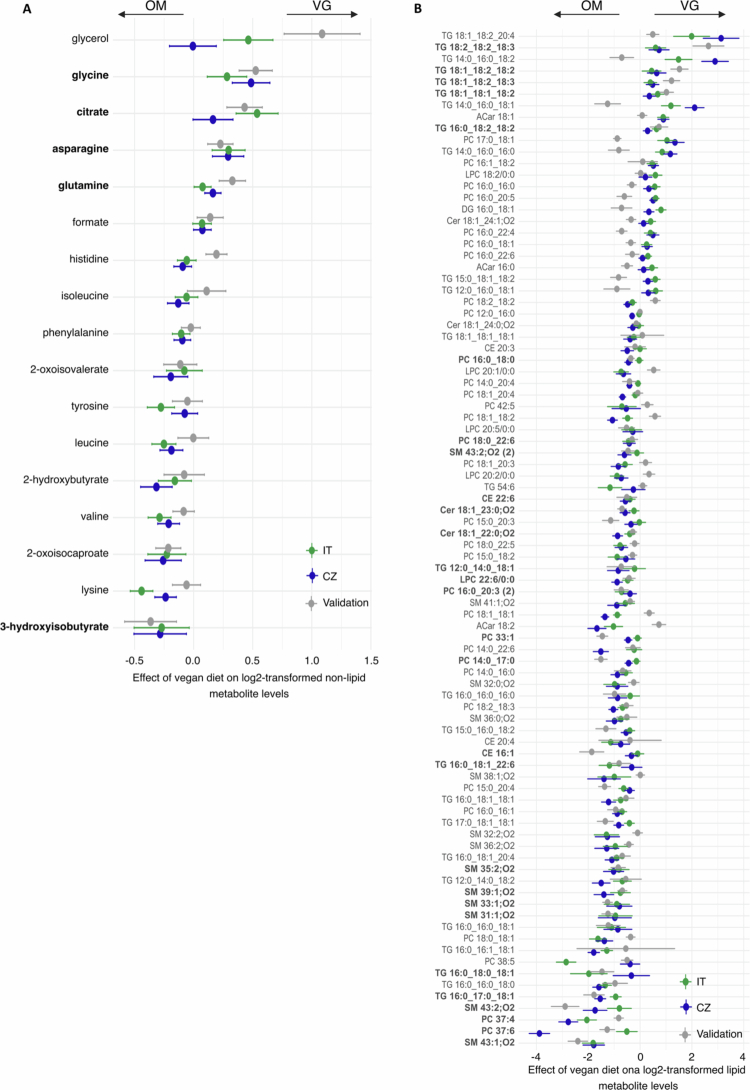
A-B. Effects of a vegan diet on the levels of selected (A) non-lipidic and (B) lipidic metabolites. The plot displays the estimated diet effects and 95% confidence intervals across all three cohorts (training CZ and IT cohorts and external validation CZ cohort), reporting only the variables that significantly differed between diets in the training cohorts. Positive estimated values/intervals of contrast indicate higher values of individual variables for the vegan group than for the omnivore group. The variables marked in bold belong to the *vegan signature.*

Identifying the vegan diet-dependent variables is not trivial because most of them may be influenced by the country of origin and the local diets of the cohorts. Therefore, we employed linear models, allowing the estimation of the effects of both main factors (diet, country) and their interaction on individual lipids, metabolites, and microbial taxa (features). Features that showed a significant diet effect (average effect of diet across both countries, adjusted for multiple comparisons with FDR<0.05) were then visualized using forest plots (Figures 3 and 4, Supplementary Table 5). We applied additional criteria to this selection to find a *vegan signature,* including the following: (i) diet-related differences in the given features remained significant in the validation dataset (*p* < 0.05); (ii) the direction of the diet effect was consistent across all three cohorts; (iii) the feature had some predictive power in the elastic net (|β| > 0.1), and the prediction direction was the same as that of the diet-related difference in the linear model.

In the microbiome dataset, 39 taxa with significant diet effects were identified by the linear model (Figure 3A, Supplementary Table 5A). Twenty-three SGBs were consistently more abundant in OM, while 16 were more abundant in VG subjects. When we applied the additional criteria described above, we identified 11 SGBs that represent the microbiome-based *vegan signature.* Among these SGBs, four (*Ruminococcus_sp_AF41_9|SGB25497, Blautia obeum|SGB4810, Roseburia lenta|SGB4957, SGB14987*) presented an increased abundance in VG, while 7 (*Youxingia wuxianensis|SGB82503, Eubacterium ramulus|SGB4959, Holdemania filiformis|SGB4046, Streptococcus thermophilus|SGB8002, Ruminococcus torques|SGB4608, Lawsonibacter saccharolyticus|SGB15154, Enterocloster hominis|SGB4721*) were more abundant in OM. Coherent differences were observed when the effect sizes of the models computed by Fackelmann and colleagues were considered^10^ for four additional independent cohorts and for the meta-analysis model (Figure 3B). We further employed ZOE MB health ranks, which assign a numeric ranking to SGBs based on their correlation with cardiometabolic health markers. The lower the value of the ZOE rank is, the more beneficial the cardiometabolic status indicates. In this respect, the SGB ranking of microbes positively associated with the omnivore diet (median rank = 0.572) was significantly higher than that of those more abundant in VG (median rank = 0.236, *p* < 0.001) (Figure 3C).

Using StrainPhlAn v4.0, we derived possible sample-specific strains of each SGB. Three SGBs (*Clostridium fessum*-SGB4705, *Lawsonibacter asaccharolyticus*-SGB15154, and *Ruthenibacterium lactatiformans*-SGB15271) were available in more than 60 samples with enough markers to construct the final phylogenetic trees. Interestingly, the analysis related to *Clostridium fessum*-SGB4705, which included the highest number of samples (*n* = 106), identified a clade that was prevalently detected in Italian omnivores (Supplementary Figure 7).

In the serum metabolome dataset, 17 nonlipid metabolites with significant diet effects were identified via the linear model (Figure 4A, Supplementary Table 5B). Six of them were higher in VG (glycerol, citrate, formate, glycine, glutamine, and asparagine), while 11 were higher in OM (2-hydroxybutyrate, lysine, histidine, tyrosine, phenylalanine, valine, leucine, isoleucine, and intermediates of branched-chain amino acid metabolism). The non-lipid metabolome-based *vegan signature* consisted of glycine, citrate, glutamine, asparagine (higher in vegans), and 3-hydroxyisobutyrate (higher in omnivores).

The VG serum lipidome (92 lipids selected by a linear model) was characterized by a lower content of lipid species containing saturated fatty acid moieties and higher representation of lipids with higher levels of saturation (Figure 4B, Supplementary Table 5C). The level of arachidonic acid bound to different types of lipids was consistently higher in omnivores. Sphingomyelins (SMs), cholesterol esters (CEs), and ceramides (CERs) are underrepresented in vegans. The profiles of phospholipids, lysophospholipids (LPCs), and triacylglycerols (TAGs) reflected the saturation status of the incorporated fatty acids. After the application of additional criteria, we identified a subset of 27 lipids representing lipidome-based *vegan signature*.

**Figure 5.**
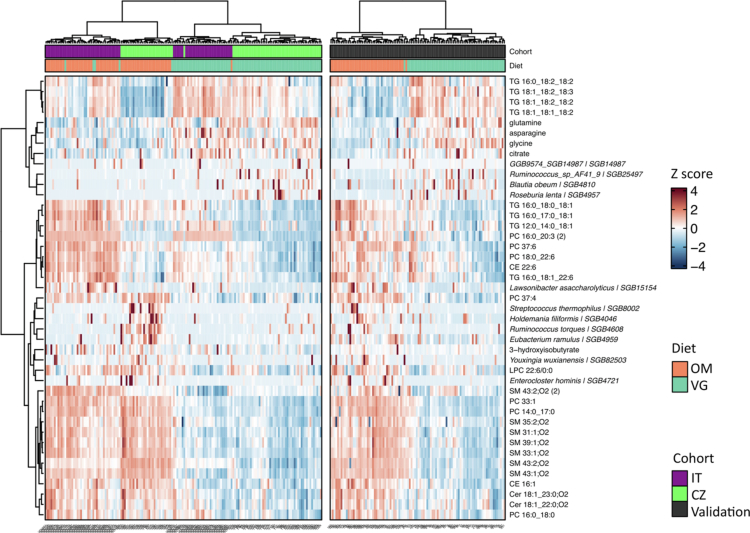
Heatmap and clustering dendrogram of samples relative to the vegan signature feature levels. The levels of each variable are reported as Z-scores. Sample annotations are colored according to the country (CZ, IT) and diet (VG, OM).

In the next step, we combined the microbiome, non-lipid metabolome and lipidome features that passed the additional criteria into the combined *vegan signature.* The dietary group classification efficiency of the combined *vegan signature* (*n* = 43) (Table 3) was evaluated by hierarchical clustering (Figure 5). The outcome proved a distinction independent of the country of origin between VG and OM across all datasets, as only four VG and one OM (2.9%) were misclassified in the training dataset, and two (1.4%) VG were incorrectly classified in the validation CZ cohort. On the other hand, within the OM in the training cohorts, there is a clear separation according to the country for some but not all variables. The country-specific pattern was observed in lipids containing unsaturated fatty acids, and five SGBs were more abundant in VG. In contrast, the distribution of SMs, CERs, non-lipid serum metabolites, and SGBs that are more abundant in VG, exhibited no country effects. Similar results were obtained using the whole set of 150 MIME features selected by GLM (Supplementary Figure 8).

**Table 3.** Variables contributing to the combined vegan signature.

	Lipids	Non-lipid metabolites		Microbes	
Higher in vegans	TG 18:2_18:2_18:3	glycine		*Roseburia lenta*|SGB4957	
	TG 18:1_18:2_18:2	citrate		*Blautia obeum*|SGB4810	
	TG 18:1_18:2_18:3	glutamine		GGB9574 SGB14987|SGB14987	
	TG 18:1_18:1_18:2	asparagine		*Ruminococcus sp AF41 9*|SGB25497	
	TG 16:0_18:2_18:2				
Higher in omnivores	SM 31:1; O2	3-hydroxyisobutyrate		*Youxingia wuxianensis*|SGB82503	
	SM 33:1; O2			*Eubacterium ramulus*|SGB4959	
	SM 35:2; O2			*Holdemania filiformis*|SGB4046	
	SM 39:1; O2			*Streptococcus thermophilus*|SGB8002	
	SM 43:1; O2			*Ruminococcus torques*|SGB4608	
	SM 43:2; O2			*Lawsonibacter asaccharolyticus*|SGB15154	
	SM 43:2; O2 (2)			*Enterocloster hominis*|SGB4721	
	Cer 18:1_22:0;O2				
	Cer 18:1_22:0;O2				
	CE 22:6				
	CE 16:1				
	PC 18:0_22:6				
	PC 16:0_18:0				
	PC 16:0_20:3				
	PC 37:6				
	PC 33:1				
	PC 14:0_17:0				
	LPC 22:6/0:0				
	TG 12:0_14:0_18:1				
	TG 16:0_18:1_22:6				
	TG 16:0_18:0_18:1				
	TG 16:0_17:0_18:1				

Cer, ceramides; CE, cholesterol esters; LPC, lysophospholipids; PC, phosphatidylcholines; SM, sphingomyelines; TG, triglycerides.

### Functional analysis of gut microbiota

Based on shotgun sequencing data, a detailed assessment of the functional capacity of the gut microbiota was performed. The median proportions of unmapped and unintegrated reads across cohorts were 20%–23% and 72%–74%, respectively. Within the classified pathways, 9%–11% of the pathway signals could not be attributed to specific taxa (“unclassified”).

**Figure 6.**
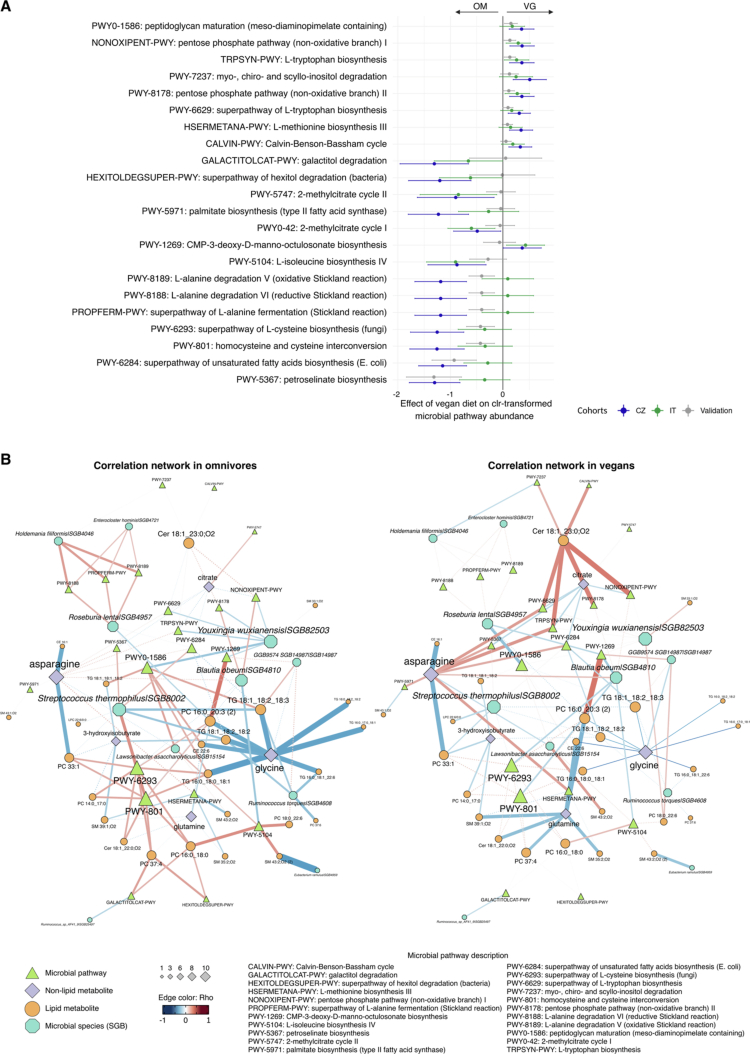
Effects of a vegan diet on the levels of microbial pathways and the vegan signature feature correlation network. (A) The plot displays the estimated diet effects and 95% confidence intervals across all three cohorts for the HUMAnN microbial pathways identified as significantly different between diets in the training cohorts. (B) Correlation network among diet-dependent microbial pathways and the vegan signature features considering the correlations computed for the omnivores (left) and vegans (right). The node size is proportional to the number of significant correlations identified, while the edge color represents the Spearman correlation coefficient. The edge width is proportional to the correlation significance, with dashed lines representing non-significant (FDR corrected *q* < 0.05) associations. The class of the different MIME features is decoded as the node shape and color.

Altogether, 350 metabolic pathways were quantified, 22 of which were significantly differentially abundant (*q* < 0.05) between VG and OM (Figure 6A). Eight pathways were uniformly enriched in VG: the pentose phosphate pathway, amino acid (tryptophane, methionine) biosynthesis, peptidoglycan maturation, and inositol degradation. In OM, increased metabolic capacity for unsaturated and saturated fatty acid biosynthesis, amino acid fermentation for energy production (Stickland reaction), BCAA (isoleucine) biosynthesis, propanoate utilization (methylcitrate cycle), cysteine biosynthesis, and homocysteine-to-cysteine interconversion were observed. Metabolic pathways that are more characteristic of OM exhibited inconsistent patterns, with considerable differences among OM cohorts.

Correlation analysis between the predicted pathway abundances and the levels of features belonging to the combined *vegan signature* showed 82 significant associations (*q* < 0.05 and coherent coefficients among the three cohorts), of which 51 were more typical in OM, 27 in VG and four in common (Figure 6B and Supplementary Table 5D). The *homocysteine and cysteine interconversion* (PWY-801) and *superpathway of L-cysteine biosynthesis* (PWY-6293) were those with the highest number of significant correlations in OM, while the *superpathway of unsaturated fatty acid biosynthesis* (PWY-6284) was the most connected pathway node in the vegan network.

We further examined the taxonomic contributors to the diet-sensitive pathways using the stratified HUMAnN3 output. In most cases, the main contribution comes from an unclassified signal, or a heterogeneous set of contributors is involved. Only a minor portion of pathways are driven by specific taxa, e.g., L-tryptophan biosynthesis (*B. vulgatus*) or the superpathway of unsaturated fatty acid biosynthesis (*E. coli*) (Supplementary Table 6).

### The effect of the duration of the vegan diet on the investigated omics

We also investigated whether the omics features that distinguished VG from OM varied with the length of adherence to the vegan diet. Across the microbiome, metabolome, and functional pathway datasets, no consistent trends were observed, and the associations detected in one training cohort were not reproduced in the validation cohort (Supplementary Table 7 and Supplementary Figure 9). However, several lipids from the lipidome data, particularly those characteristic of an omnivorous diet, showed decreasing levels with longer adherence to the vegan diet. Two sphingomyelins (SM 33:1;O2 and SM 43:1;O2), which are strongly associated with the omnivorous diet, also displayed significant negative associations with vegan diet duration in the training cohort (*P*-value for the average effect <0.05), and these findings were validated in the independent cohort. Five additional lipids (PC 14:0_17:0, PC 15:0_20:3, PC 37:6, SM 36:0;O2, TG 16:0_18:1_22:6) showed consistent negative associations but did not reach statistical significance in the validation cohort. Overall, these results suggest that some lipids typical of an omnivorous diet may gradually decrease with increasing vegan duration.

### Relationship between diet-specific omics patterns and metabolic health markers

Finally, we investigated the associations between clinical markers of metabolic health and the combined *vegan signature* features identified here (Supplementary Figure 10). Serum TC, LDL-c, triglycerides, and BMI inversely correlated with several *vegan signature* components that were more abundant in vegan cohorts, i.e. non-lipid serum metabolites (glutamine, glycine, asparagine, citrate) and bacteria (*R. lenta|SGB4957, B. obeum|SGB4810).* In contrast, a positive correlation was found between these variables and the omnivore lipidome signature, i.e., higher SMs, CERs, lipids containing saturated or Ѡ-3 unsaturated fatty acids, or omnivore-characteristic bacterial taxa (*L. asaccharolyticus, Youxingia wuxianensis, R. torques, S. thermophilus*, *Enterocloster hominis*).

## Discussion

Here, we report findings from a cross-sectional study aimed at investigating the multi-omics profiles of two geographically distinct populations, VG and OM. As the major outcomes of the study, we: (1) described specific omics profiles (lipidomic, metabolomic, and metagenomic) distinguishing the dietary groups and (2) reported the high discriminative power of the identified *vegan signature*, which was also confirmed in an independent validation cohort. Our observations suggest that, in addition to identifying distinct regional traits associated with diets, it is possible to identify multi-omic features that characterize a specific diet independently of its country of origin.

### Vegan diet is associated with a specific gut microbiota profile

Previous studies, often based on cultivation methods or less advanced sequencing techniques, demonstrated only modest differences between vegan and omnivore gut microbiota.^5^^,^^8^^,^^22^ In contrast, using shotgun sequencing, we and others have unraveled a characteristic vegan-specific microbiota profile. Indeed, our findings align with those of a recently published study on 656 VG and 19,817 OM in which 30 top SGBs associated with the dietary pattern were identified, with 11 taxa being more abundant in VG and 19 in OM.^10^ Hereby, we reported 39 diet-associated SGBs, 14 of which overlapped between both studies. These results confirm that a vegan diet is associated with a distinct composition of the gut microbiota, different from that of omnivores but also independent of country of origin.

Although the precise metabolic activity of the identified taxa and their association with health cannot be generalized, it was interesting to compare the vegan signature SGBs identified in our study to the recently published ZOE Microbiome Ranking, which assigns a numeric ranking to SGBs found to significantly correlate with cardiometabolic markers significantly. Based on this criterion, SGBs more abundant in OM resulted in a higher rank (i.e., less beneficial) than those more abundant in VG.

Several taxa found to be less abundant in vegans in our study have previously been associated with adverse health outcomes, including cardiometabolic and inflammatory diseases.^23-28^ Conversely, taxa promoted in vegans are often related to high fiber intake, healthier dietary patterns, and beneficial health markers.^10^^,^^29^ However, these results should be interpreted with caution, as they may reflect underlying dietary contexts typical of disease rather than direct causal effects of the microbes themselves. Overall, our findings illustrate how a vegan diet-associated shift in the gut microbiota may contribute to host health, complementing the observed alterations in the serum lipid metabolome.

### Vegan and omnivore microbiome is associated with different keystone taxa

The concept of keystone species facilitating community stability and resilience originates in macro-ecology.^30^^,^^31^ Aiming to identify such keystone taxa in the human gut microbiome, Bauchinger and colleagues,^32^ using publicly available metagenome data of healthy subjects, identified several genera with keystone potential, including *Holdemania* and *Agathobaculum.* Interestingly, we identified the former as significantly enriched in OM microbiomes, while the latter was characteristic of VG. *Holdemania*, the keystone genus of the omnivore microbiome, has been associated with processed meat intake^33^ and obesity in adults.^34^ On the other hand, the butyrate producer *Agathobaculum*, the keystone genus of the vegan microbiome, are associated with increased microbiome stability in travelers,^35^ increased fiber intake,^29^ and a protective effect against neurodegenerative diseases.^36^ Furthermore, *Holdemania* and *Agathobaculum* clusters identified in our study partially overlap with the work of Bauchinger and colleagues.^32^ This finding supports the idea that diet distinctly shapes the whole gut microbiome ecosystem.

### Functional capacity of the microbiome reflects the diet approach

Substantial evidence has demonstrated that diet significantly influences the functional capacity of the gut microbiota, primarily through shifts in microbial metabolism and metabolite production. In agreement with previously published studies,^10^^,^^37^^,^^38^ we observed a specific pattern of metabolic adaptation to the prevalent energy source in the metagenomes of VG and OM. The omnivore metagenome was enriched in pathways that allowed for amino acid fermentation, which might reflect the higher availability of amino acids in the distal intestine associated with the omnivore diet. The omnivore microbiome seems to be better equipped for homocysteine/cysteine interconversion and cysteine biosynthesis,indicating a difference in sulfur metabolism compared to VG. Finally,we found a higher capacity for the degradation of hexiols (mannitol, sorbitol, galactitol) in the omnivore microbiome, which might indicate higher intake of artificial sweeteners in this dietary group. On the other hand, the vegan metagenome was enriched in bacteria specialized in amino acid biosynthetic pathways, particularly tryptophan and methionine, which might be underrepresented in a vegan diet.^39^ The bacteria associated with inositol degradation pathways were also more abundant in the VG. Since the primary sources of dietary inositol are fruits and vegetables, this observation may be a reflection of the vegan diet, considering that commensal bacteria widely use myo-inositol as a source of carbon and energy.^40^

### The vegan diet is associated with a specific lipid serum profile different from omnivore diet

The lipidome differed markedly between VG and OM and was the most distinctive feature separating the two dietary groups independently of country of origin. Vegans showed lower levels of SMs, CERs, and CEs but higher levels of unsaturated fatty acids. Furthermore, some lipids associated with the omnivore diet exhibited an inverse relationship with the duration of the vegan diet (i.e., SM 33:1;O2, SM 43:1;O2). SMs, which are abundant in animal-derived foods and low in plants,^41^^,^^42^possess pro-atherogenic properties, and plasma SM levels are an independent risk factor for coronary heart disease.^43-45^ As direct precursors of CERs, they are linked to key processes in atherosclerosis,^46^and elevated CERs are associated with coronary artery disease and cardiovascular mortality (BECAC and SPUM-ACS studies).^47^ In contrast, omnivores presented higher levels of CE (16:1), a major plasma cholesterol ester whose arterial accumulation is a hallmark of atherosclerosis and obesity, and has been linked to cardiovascular disease progression.^48^

An extensive review based on 16 lipidomic studies reported that, independent of HDL-c and LDL-c serum concentrations, phosphatidylcholines containing saturated and monounsaturated fatty acyl chains were associated with an increased risk of cardiovascular disease, while phosphatidylcholines containing polyunsaturated fatty acyl chains were inversely associated with it.^49^ In the current study, the vegan diet was more associated with phosphatidylcholines containing polyunsaturated fatty acids than the omnivore dietary pattern. Furthermore, PCs containing oleic and linoleic acids were positively correlated with HDL-c while having no association with LDL-c or TC.

### Vegan metabolome is associated with better indices of cardiometabolic health

The serum non-lipid metabolite signature associated with the vegan diet in this study was almost opposite that associated with the metabolome, which is generally related to obesity, metabolic syndrome, and type 2 diabetes (T2D). High serum concentrations of lysine, phenylalanine, BCAA, and their intermediates and low concentrations of citrate, glycine, glutamine, and asparagine are among the characteristic patterns of the metabolic challenge. Phenylalanine and BCAA were identified as predictors of an increased risk of T2D development,^49^^,^^50^ whereas glycine and asparagine are markers of low risk for the disease.^50-52^

Some features characterizing the vegan metabolome profile (high citrate, low 2-oxoisocaproate, decreased valine and leucine, as well as increased glycine) mirrored the metabolomics signatures of metabolic health, contrasting with NCD-associated profiles.^46^^,^^53-56^ Our previous study on the Czech subjects of the training cohort^5^ revealed a higher content of SCFA in feces and serum of VG, which is consistent with the observed differences in microbiota composition and may also help explain the beneficial effect of a vegan diet on gut health.

Amino acid serum composition is typically considered a sole function of the host metabolism, but recent research has provided novel insights into the role of the gut microbiota in amino acid homeostasis. Serum glycine has been repeatedly shown to be higher in VG despite lower dietary intake. Carter et al. demonstrated that some gut bacteria metabolize glycine, whose elevated serum concentration is a strong predictor of vegan status, and thus modulate its availability for the host.^57^ These relationships may represent new mechanisms by which the gut microbiota regulates host metabolic status through diet.

### Country of origin influences the MIME profiles in omnivores but not in vegans

Identifying diet-specific omics signatures across different countries is challenging because of significant geographical bias. Although we addressed this issue in the data analysis, an uneven distribution of some diet-dependent variables by geography remained, particularly in the OM groups. Concerning the serum lipidome, the Italian omnivore lipidome profile was enriched in triglycerides containing oleic, linoleic, and *α*-linolenic acids, as well as phospholipids containing Ѡ-3 fatty acids, which probably reflected the differences between Mediterranean and standard central European eating habits and food preparation. In contrast, lipid markers related to animal product consumption, like SMs, CERs, or CEs, were similarly abundant in the Czech and Italian OM. Some omnivore-characteristic bacteria, *S. thermophilus|SGB8002, R. torques|SGB4608, and L. asaccharolyticus|SGB15154*, also exhibited uneven distributions among the Czech and Italian OM, the former being more abundant in the Czech cohort, while the latter being more represented in the Italian cohort. *S. thermophilus|SGB8002* and *R. torques|SGB4608* might be associated with increased dairy and red meat intake, which is typical for a standard European diet. The abundance of *L. asaccharolyticus|SGB15154* is associated with coffee consumption.^58^ In contrast, no significant clustering by country was observed among vegan diet-associated features. While the omnivore diet reflects regionally specific characteristics, the dominant unifying determinant of the effects of a vegan diet is the absence of animal products.

### Strengths and limitations

The study has several key strengths: (1) it was performed on two reasonably large independent groups from geographically different locations; (2) the combined multi-omics approach allowed the definition of a complex vegan MIME profile; and (3) the results were validated on an independent cohort. However, limitations need to be noted. Despite our efforts to preserve homogeneity between study groups and centralize the omics analyses, the two country-specific groups were recruited independently, using different protocols. Particularly, there were differences in dietary intake analyses (prospective record vs FFQ). The FFQ used in IT groups has not been validated for use in vegans, which could have led to an underestimation of the nutrient intake of some vegan-specific foods. The correlations between the vegan signature components and metabolic health markers suggest a potential link, but the cross-sectional design of the study does not establish causality. We cannot exclude the possibility that our findings may have also been confounded by other intrinsic factors (lifestyle habits, genetic background) affecting health outcomes independently of vegan status. Although addressed in our study, the regional effect may be more pronounced in geographically more distant populations.^59^ In addition, in the present study, the use of StrainPhlan presents some limitations since it relies on the most abundant organisms, therefore restricting a comprehensive view of strain-level dynamics if there is not a minimum coverage of species-specific markers to perform SNP analysis. Additionally, the use of the Humann3 method can be an additional limitation since, currently, it only classifies a relatively small proportion of the metagenomic data, limiting the depth of functional insights. In this context, a deeper depth of sequencing in parallel with assembly-based methods would overcome such problems allowing a more comprehensive investigation of microbial communities characterizing dietary habits across populations.

## Conclusions

In conclusion, this comparative multi-omics study of Italian and Czech VG and OM revealed that, despite country differences, a distinct vegan-associated MIME profile emerged, primarily linked to dietary patterns.

A low abundance of sphingomyelins, ceramides, cholesterol esters, and certain phosphatidylcholines, along with a higher content of glycine, glutamine, asparagine, and citrate, are characteristic features of the vegan serum metabolome. The vegan microbiome is characterized by a reduced representation of taxa inversely correlated with cardiometabolic health markers, including *R. torques, L. asaccharolyticus, Hydrogenoanaerobacterium saccharovorans* or *Enterocloster hominis*. The results confirmed that transitioning to a more plant-based diet may contribute to the prevention of cardiometabolic diseases and promote overall metabolic health.

## Supplementary Material

Supplementary material Supplementary material.

Supplementary material Supplementary material.

Supplementary material Supplementary material.

Supplementary material Supplementary material.

Supplementary material Supplementary material.

Supplementary material Supplementary material.

Supplementary material Supplementary material.

Supplementary material Supplementary material.

Supplementary material Supplementary material.

Supplementary material Supplementary material.

Supplementary material Supplementary material.

Supplementary material Supplementary material.

Supplementary material Supplementary material.

Supplementary material Supplementary material

Supplementary material Supplementary material

Supplementary material Supplementary material.

Supplementary material Supplementary material.

Supplementary material Supplementary material.

## Data Availability

Sequencing data are available in the European Nucleotide Archive database under the accession numbers PRJNA1194741 (CZ and validation cohorts) and PRJNA721545 (IT cohort). The publication of the dietary and metabolomics data is not possible, as they were not covered by the participants’ informed consent used for the study. However, the corresponding authors will make pseudonymized data available upon reasonable request.
