# Supplementary figures and images for: A vegan diet signature from a multi-omics study on different European populations is related to favorable metabolic outcomes
Source: Gut Microbes. 2025 Dec 4;17(1):2593050. doi: 10.1080/19490976.2025.2593050 (PMC12688234; doi:10.1080/19490976.2025.2593050)

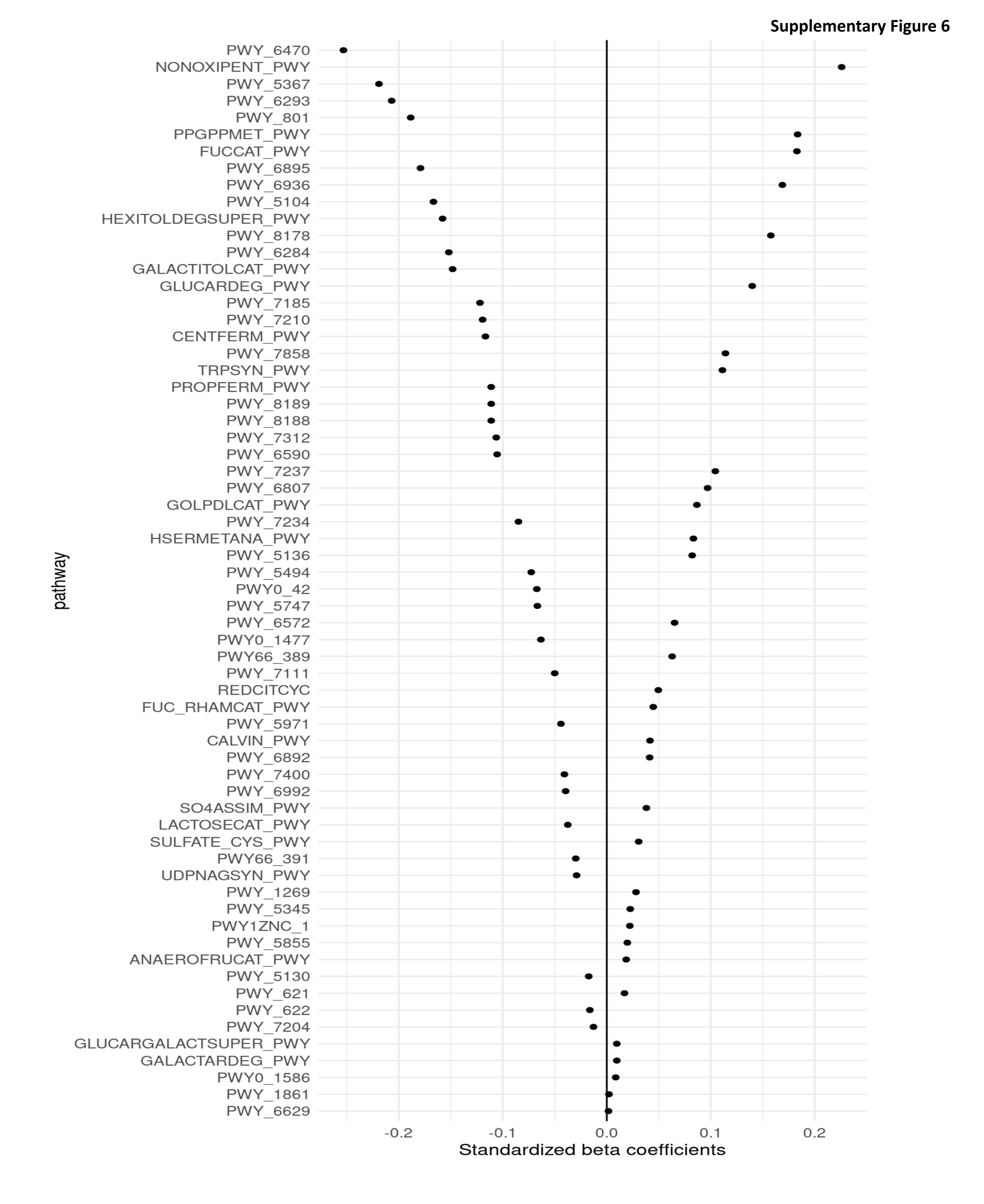

Supplement: Supplementary material [file KGMI_A_2593050_SM5034.tiff]

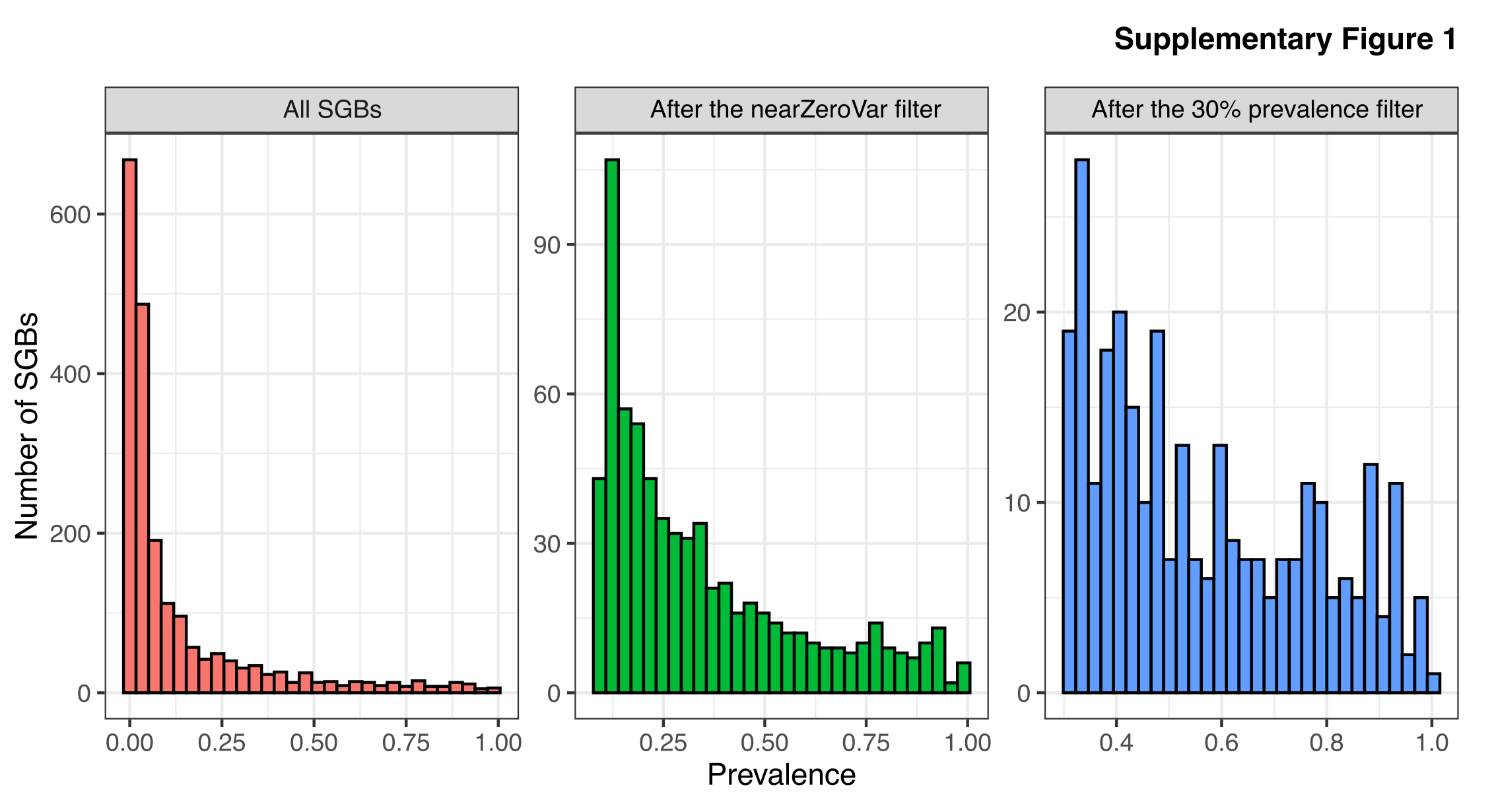

Supplement: Supplementary material [file KGMI_A_2593050_SM5035.tiff]

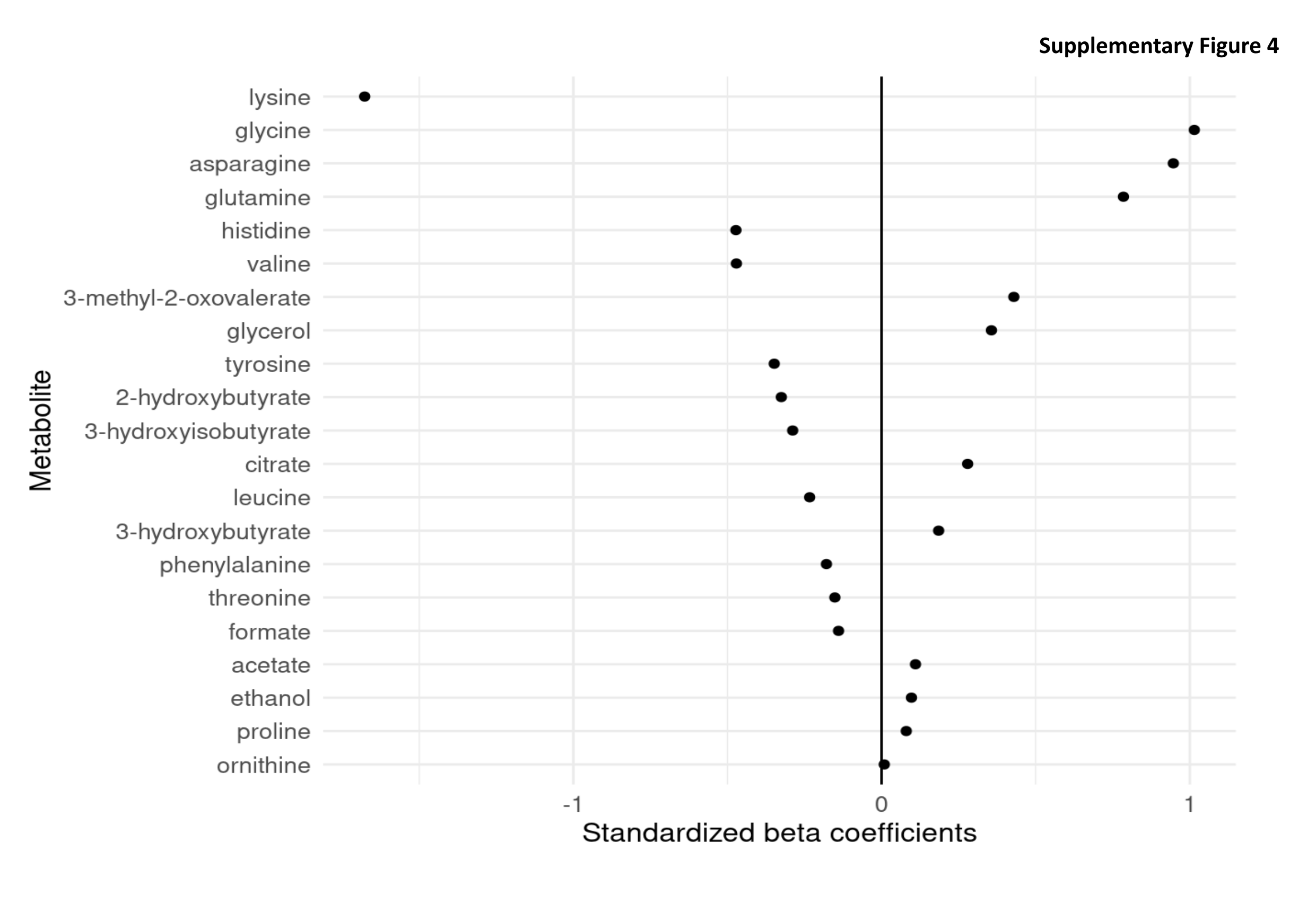

Supplement: Supplementary material [file KGMI_A_2593050_SM5038.tiff]

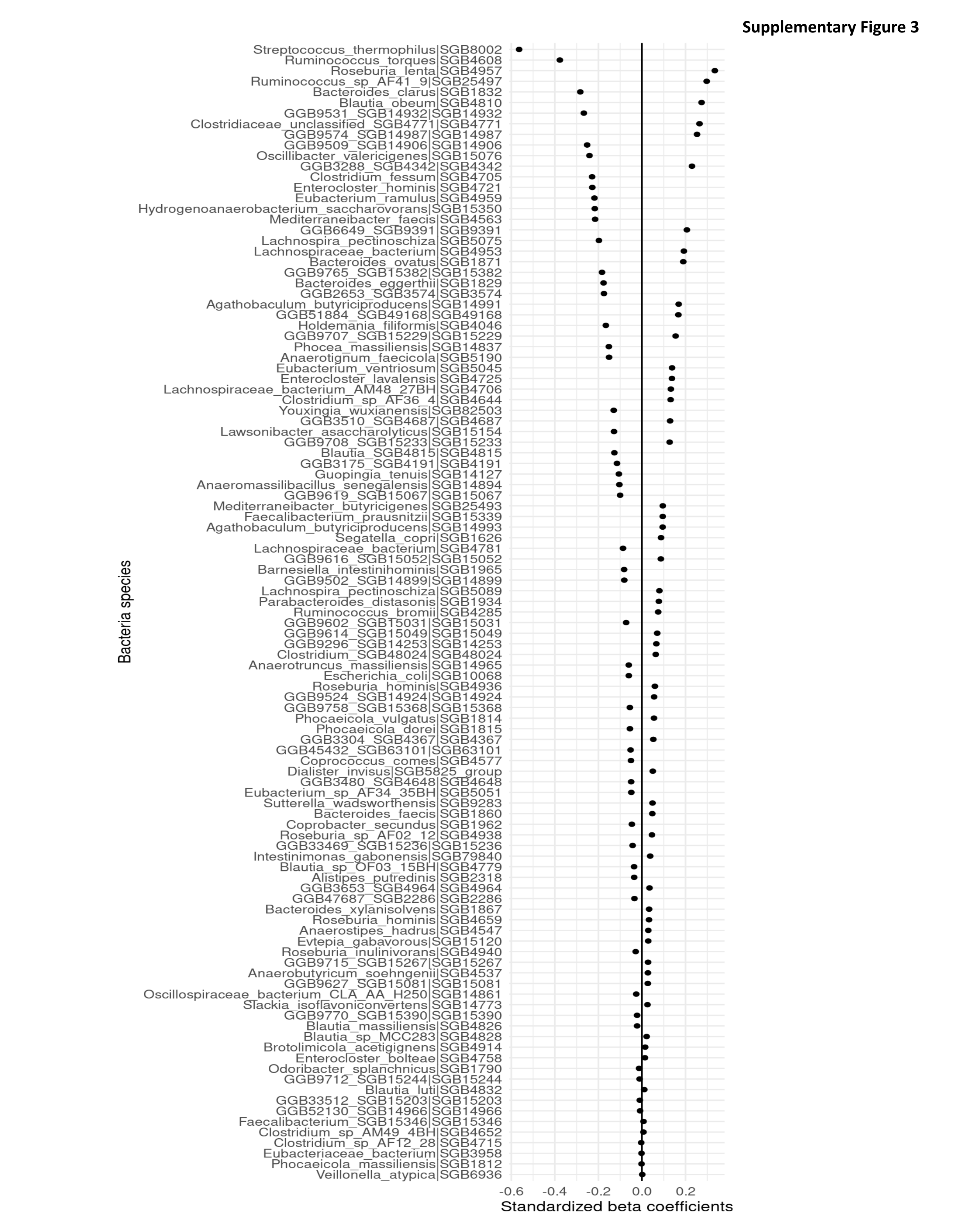

Supplement: Supplementary material [file KGMI_A_2593050_SM5041.tiff]

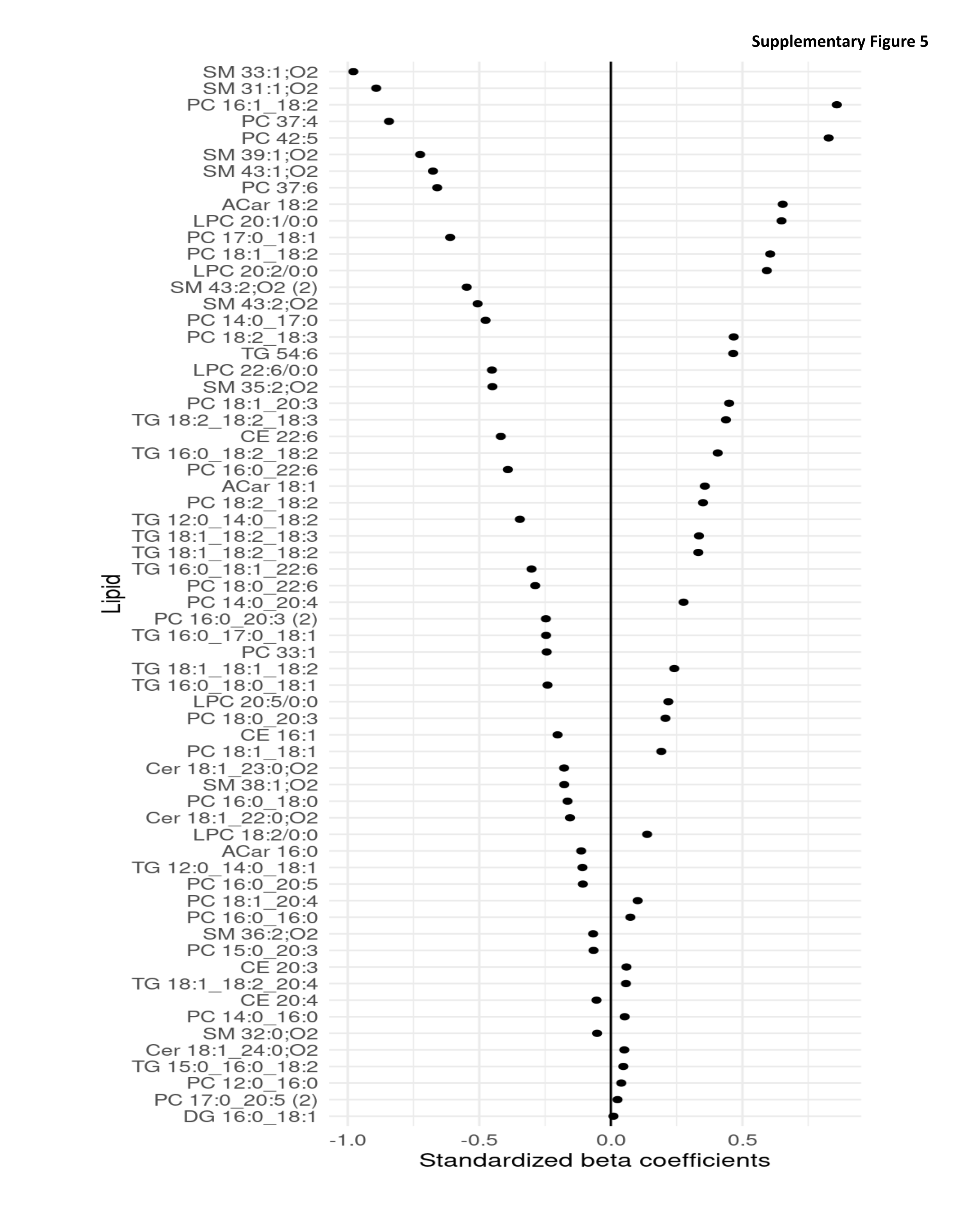

Supplement: Supplementary material [file KGMI_A_2593050_SM5042.tiff]

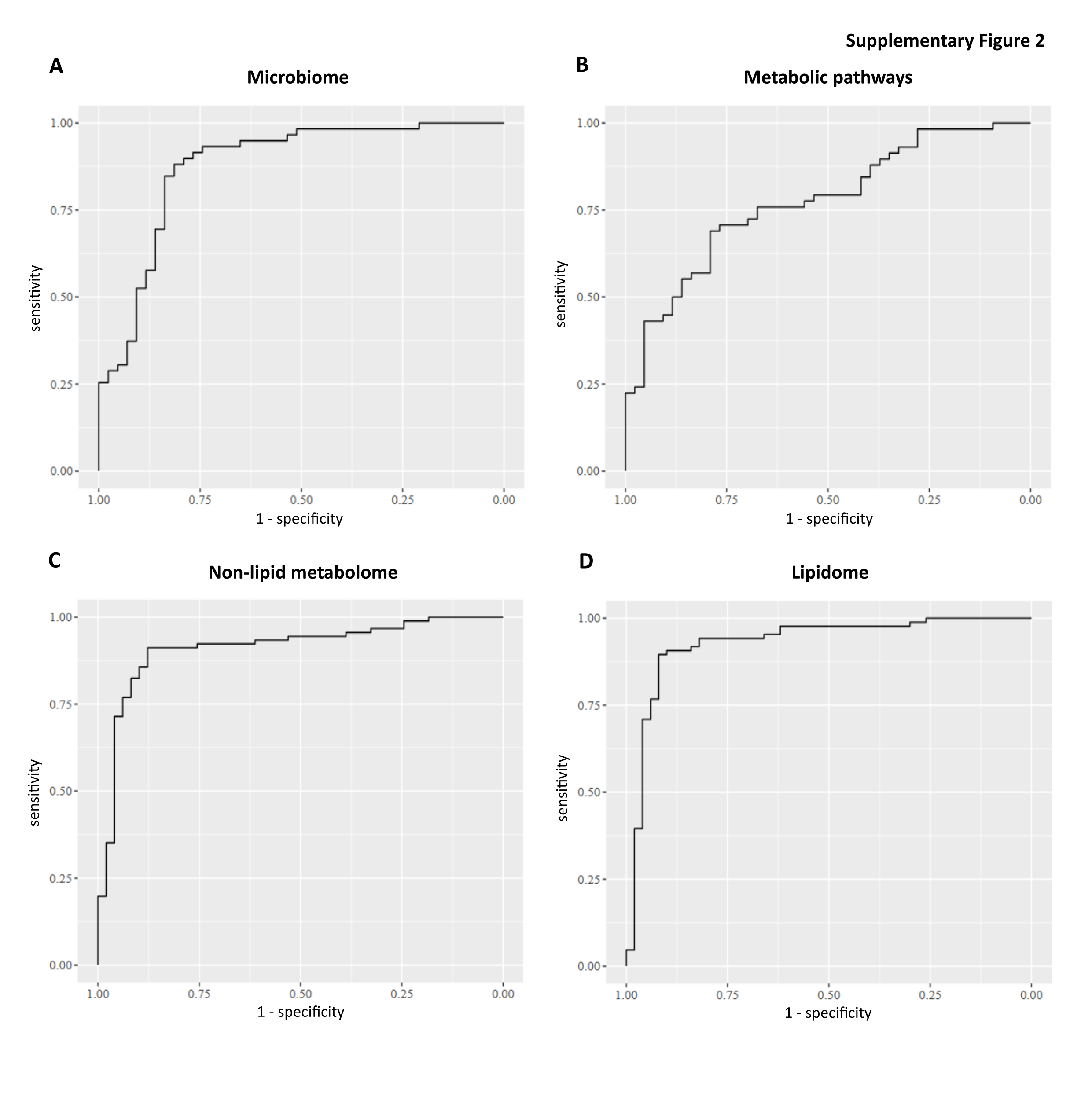

Supplement: Supplementary material [file KGMI_A_2593050_SM5043.tiff]

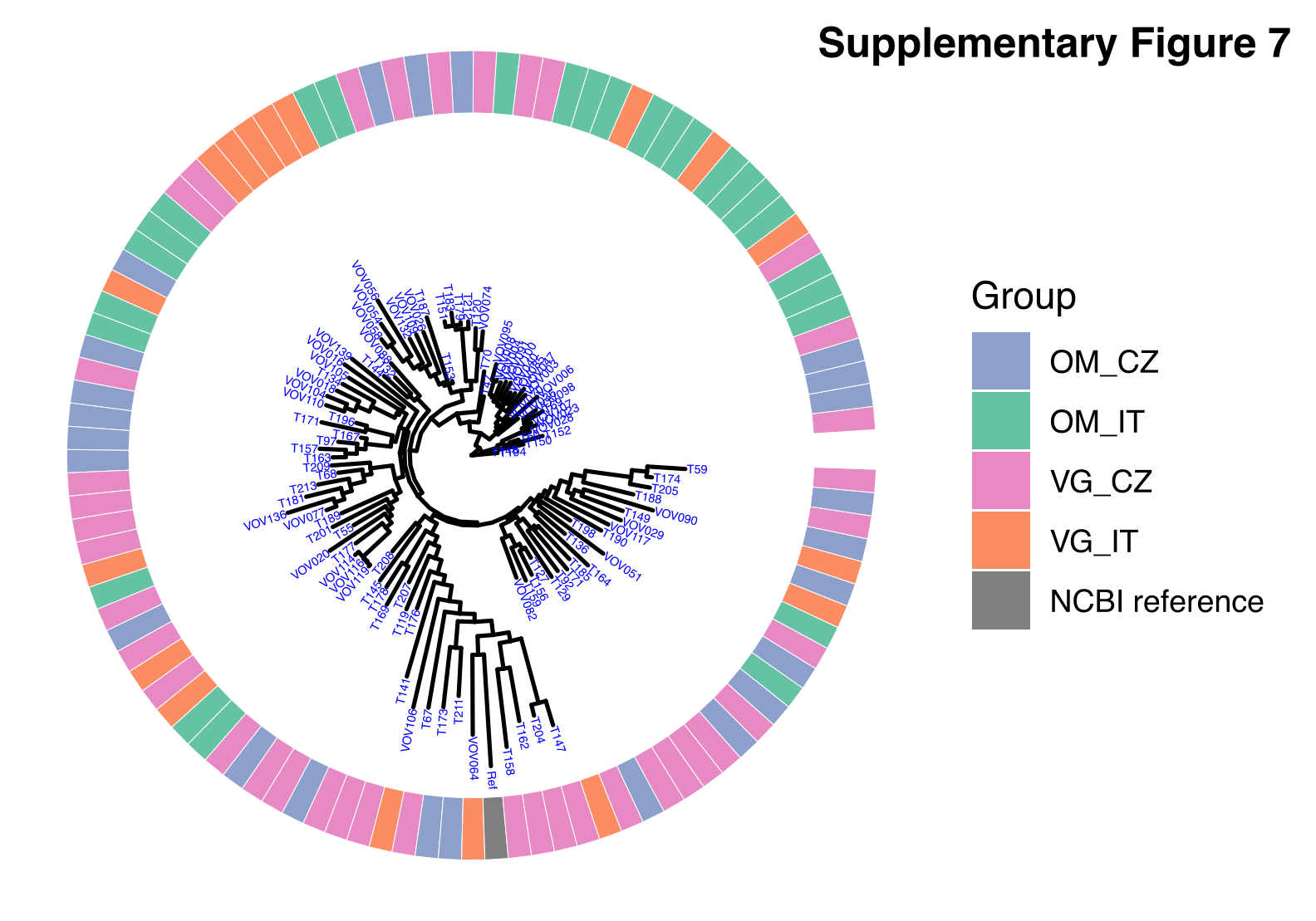

Supplement: Supplementary material [file KGMI_A_2593050_SM5044.tiff]

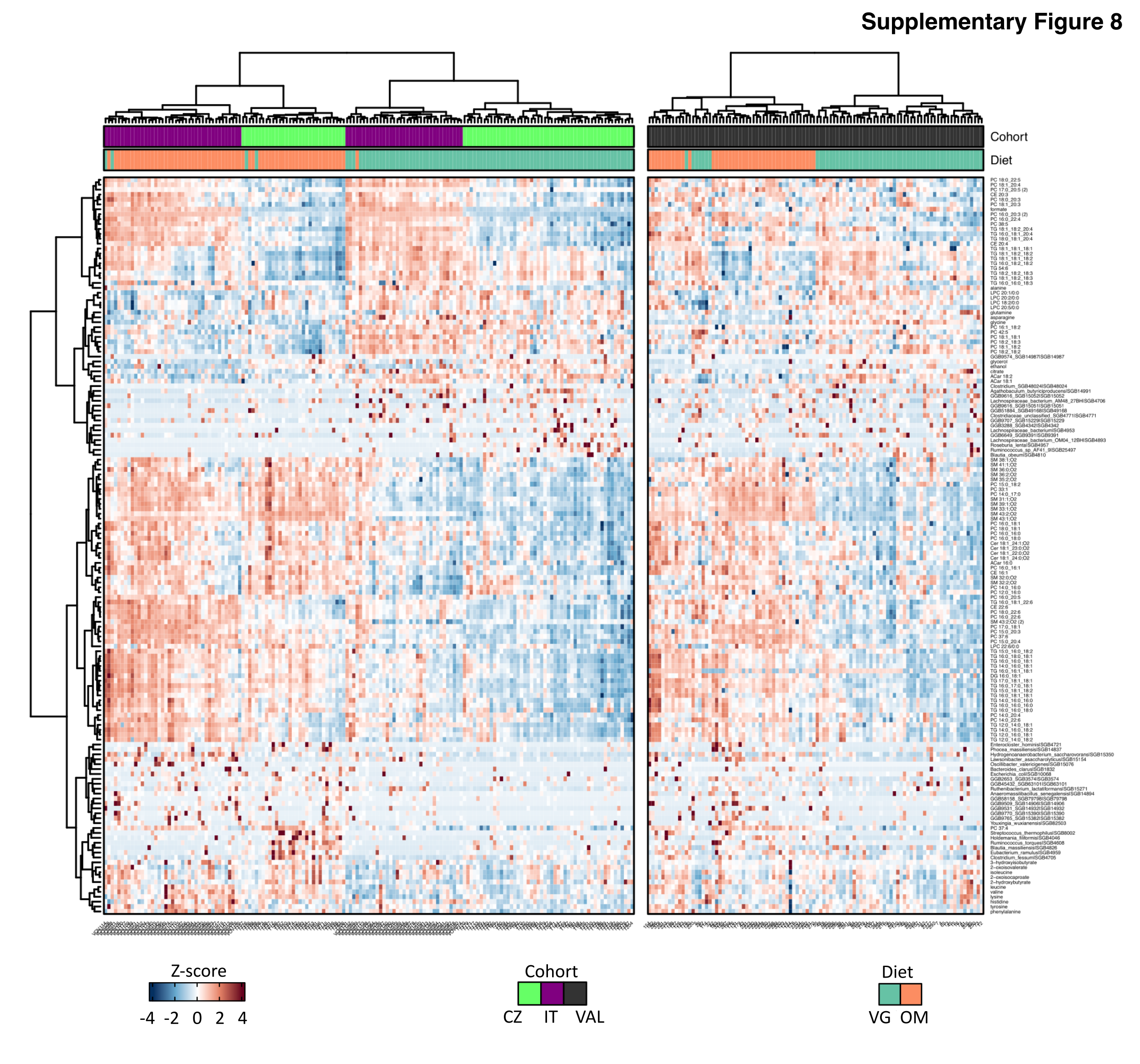

Supplement: Supplementary material [file KGMI_A_2593050_SM5048.tiff]

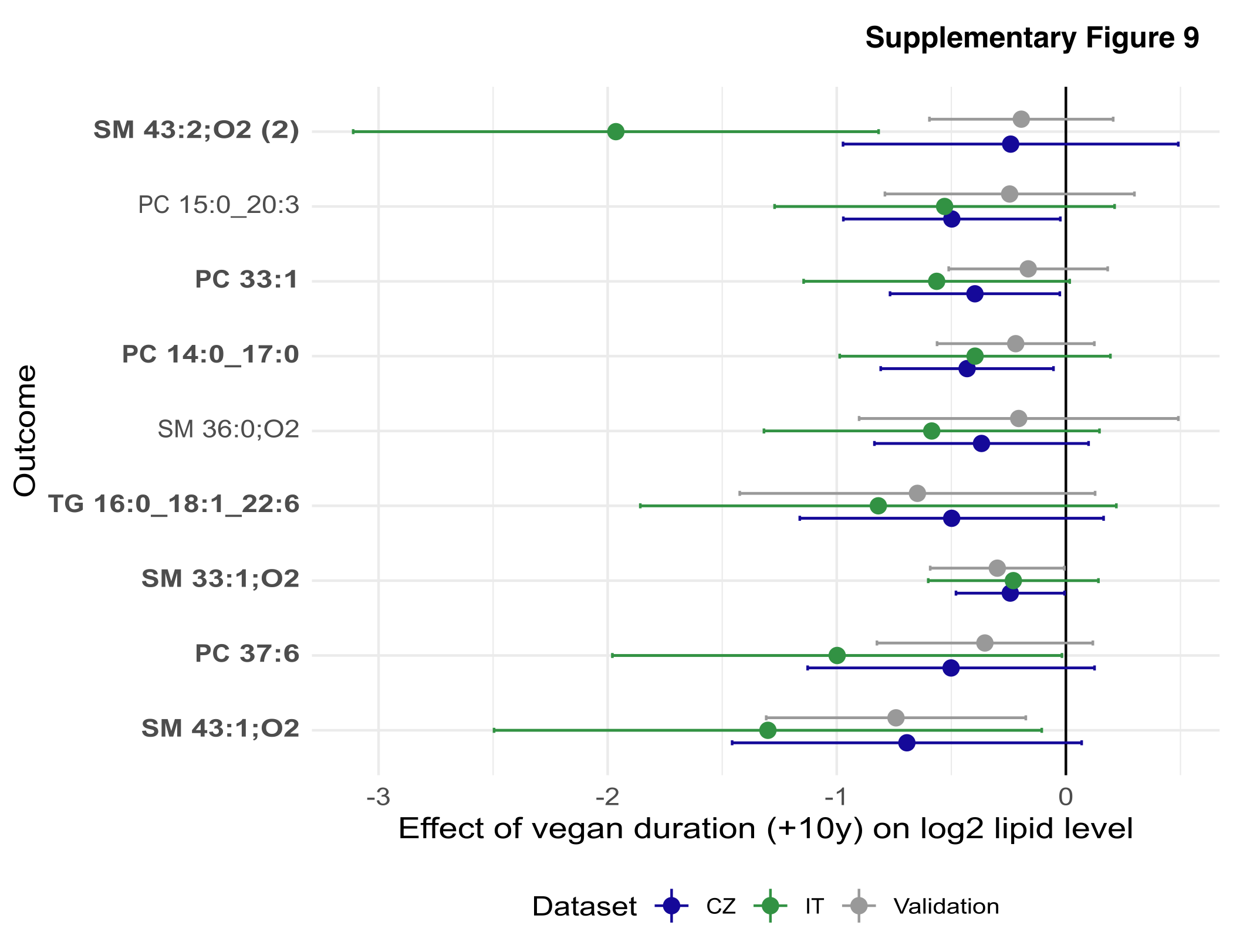

Supplement: Supplementary material [file KGMI_A_2593050_SM5049.tiff]

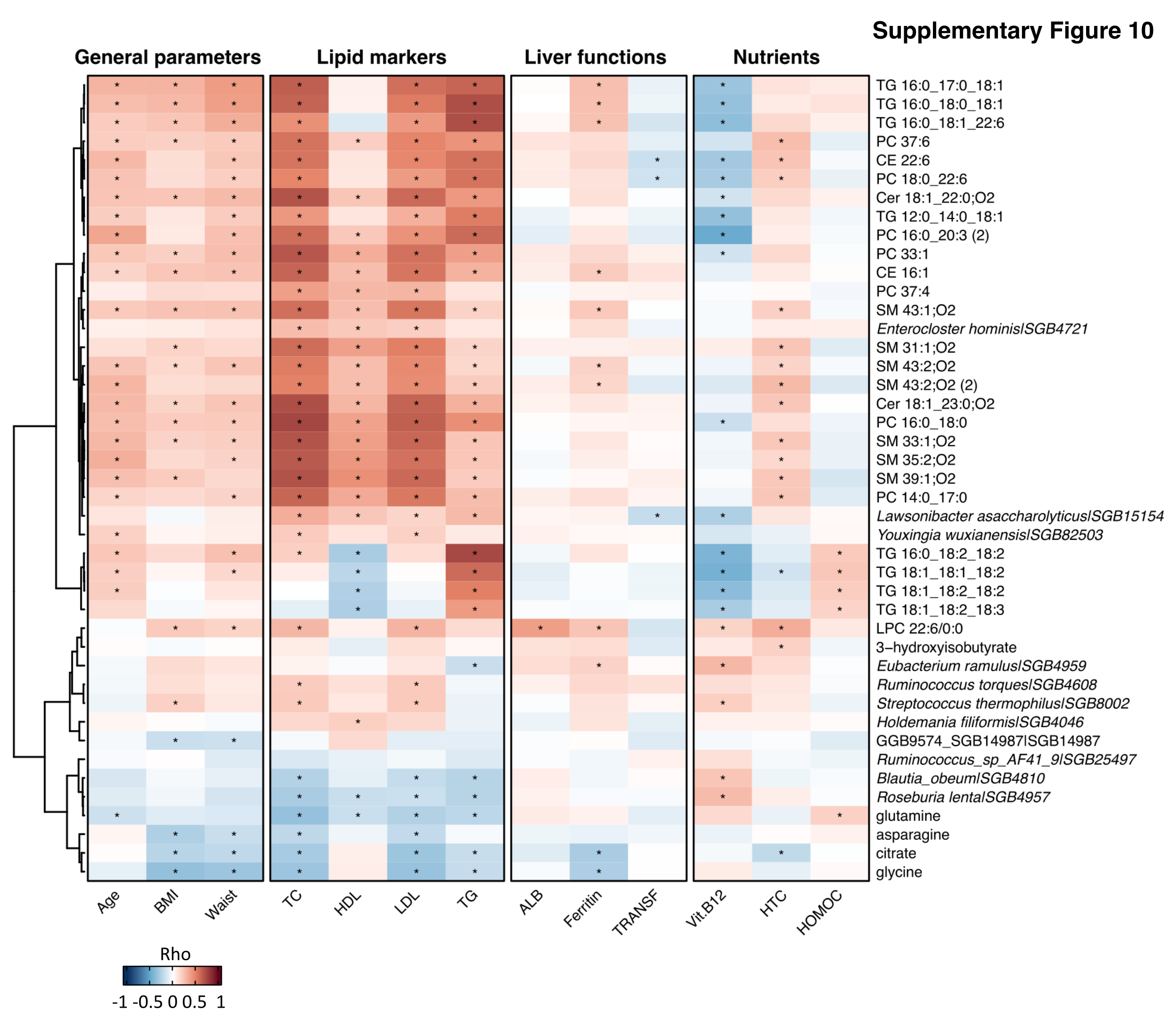

Supplement: Supplementary material [file KGMI_A_2593050_SM5050.tiff]
