# Supplementary material for: A vegan diet signature from a multi-omics study on different European populations is related to favorable metabolic outcomes
Source: Gut Microbes. 2025 Dec 4;17(1):2593050. doi: 10.1080/19490976.2025.2593050 (PMC12688234; doi:10.1080/19490976.2025.2593050)
Supplement: Supplementary material [file KGMI_A_2593050_SM5037.docx]

**Supplementary Table 2.** Group characteristics for vegans and omnivores: validation CZ cohort.

|  | **VG_CZ** | **OM_CZ** | **p-value** |
| --- | --- | --- | --- |
| **General characteristics** | | | |
| Sex [F/M] | 47/45 | 25/25 |  |
| Age  [years] | 33.9 (5.8) | 35.8 (7.8) | 0.035 |
| BMI  [kg. m^-2^] | 22.7 (4.8) | 24.5 (4.4) | 0.021 |
| Waist circumference [cm] | 76.5 (14) | 80.5 (14.3) | 0.045 |
| **Lipid metabolism** | | | |
| TC  [mmol. L^-1^] | 4.15 (1.05) | 4.85 (1.14) | < 0.001 |
| HDL-C  [mmol. L^-1^] | 1.43 (0.46) | 1.53 (0.53) | 0.496 |
| LDL-C  [mmol. L^-1^] | 2.23 (0.86) | 2.81 (0.91) | < 0.001 |
| Triacylglycerols [mmol. L^-1^] | 0.74 (0.49) | 0.87 (0.62) | 0.193 |
| **Iron metabolism** | | | |
| Ferritin  [ng. mL^-1^] | 26.6 (24.7) | 37.6 (62.0) | 0.001 |
| Transferrin  [g. L^-1^] | 2.70 (0.46) | 2.71 (0.48) | 0.461 |
| **Inflammation** | | | |
| CRP  [mg. L^-1^] | 3.99 (0.00) | 3.99 (0.00) | 0.402 |
| **Macronutrient intake** | | | |
| Total energy  [kcal. day^-1^] | 2247.0 (934.0) | 2029.0 (696.0) | 0.068 |
| Total lipids  [g. day^-1^] | 71.9 (36.1) | 77.25 (33.1) | 0.998 |
| Proteins  [g. day^-1^] | 75.5 (32.7) | 84.0 (32.9) | 0.122 |
| Carbohydrates  [g. day^-1^] | 284.3 (110.9) | 214.1 (75.5) | < 0.001 |
| Sugars  [g. day^-1^] | 65.4 (30.6) | 61.5 (42.9) | 0.668 |
| Dietary fiber  [g. day^-1^] | 37.6 (22.7) | 20.3 (10.5) | < 0.001 |
| Cholesterol  [mg. day^-1^] | 0.1 (1.1) | 163.9 (99.2) | < 0.001 |
| SFA  [g. day^-1^] | 13.9 (9.8) | 30.5 (14.3) | < 0.001 |
| MUFA  [g. day^-1^] | 20.7 (14.9) | 20.7 (12.7) | 0.765 |
| PUFA  [g. day^-1^] | 14.9  (9.3) | 8.15 (5.2) | < 0.001 |

Data are given as median (interquartile range). The overall difference among groups was evaluated using Kruskal-Wallis test followed by Mann-Whitney U test. BMI, body mass index; CRP, C-reactive protein; HDL-C, high density lipoprotein–cholesterol; LDL-C, low density lipoprotein–cholesterol; MUFA, monounsaturated fatty acids; PUFA, polyunsaturated fatty acids; SFA, saturated fatty acids; TC, total cholesterol.
