# Supplementary material for: A vegan diet signature from a multi-omics study on different European populations is related to favorable metabolic outcomes
Source: Gut Microbes. 2025 Dec 4;17(1):2593050. doi: 10.1080/19490976.2025.2593050 (PMC12688234; doi:10.1080/19490976.2025.2593050)
Supplement: Supplementary material [file KGMI_A_2593050_SM5045.docx]

**Supplementary Materials**

***DNA extraction and gut microbiome analysis***

Bacterial DNA was isolated from stool samples using the QIAamp PowerFecal Pro DNA Kit (Qiagen), and the composition of the microbiome was determined by whole metagenome shotgun sequencing. The library was prepared using the Illumina DNA Prep– (M) Tagmentation Kit (Illumina) as described in the manufacturer´s instructions starting with 24ng DNA of input in 30 µl of nuclease-free water. The bead-linked transposomes (BLT) (20 µl with one buffer/per sample) were used to fragment and tag the DNA at 55°C for 15 minutes. This step was followed by a post-tagmentation clean-up by adding Tagment Stop Buffer (TSB, 10 µl) to wash the adapter-labeled DNA on the BLT prior to PCR amplification. The sample plate was placed on the magnetic stand, the supernatant was removed and discarded, and washed twice with tagment wash buffer (TWB, 2x 100 µl). EPM and plates with unique index i5 and i7 combinations (UDC) were used for the PCR master mix (MM). The sample plate was removed from the magnetic stand, and 40 µl of MM and 10 µl of pre-paired i7 and i5 index adapters were immediately added directly to the beads in each sample well. The total PCR reaction volume was 50 µl and the amplification program included initial denaturation at 68°C for 3 min and 98°C for 3 min, followed by 8 cycles of: 45 sec denaturation at 98°C; 30 sec annealing at 62°C; 2 min elongation at 68°C; final elongation for 1 min at 68°C. After the PCR reaction, the double-sided bead purification procedure was used to purify the amplified libraries. The sample purification beads (SPB) were used twice. First, the sample plate was placed on the magnetic stand, and 45 µl supernatant from each well was transferred to a new midi plate, and 40 µl nuclease-free water and 45 µl SPB were added to the supernatant. Second, the midi plate was placed on the magnetic stand, 125 µl of supernatant was transferred to a new midi plate, and 15 µl of SPB was added to each well. Next, the supernatant was discarded, and SPB with fragments caught by the magnet were washed with 80% ethanol (2 x 200 µl). Resuspending buffer (RSB, 32 µl) has been added to the beads for the final elution.

The purified libraries were quantified using a fluorometric-based method (Quant-It or Qubit), and the library quality was checked on the Agilent Fragment Analyser 5200 or 2100 Bioanalyser (Agilent) using appropriate High Sensitivity DNA kits. All libraries were pooled to a total molarity of 150nM. The final library was sequenced on an Illumina NovaSeq6000 sequencer (Illumina). The read preprocessing (adapter trimming and removal of low-quality reads), the alignment on PhiX control, and the human genome (hg38) were performed as described in the study by Thomas et al.^1^, using the pipeline available at https://github.com/SegataLab/preprocessing.

Taxonomic profiling was performed with MetaPhlAn 4.1^1 2^ and the ChocoPhlAn vJun23 database. MetaPhlAn was applied in the default setting with a stat_q value equal to 0.1. Microbial pathway abundances were estimated using HUMAnN 3.9 applied in default settings and using the full UniRef90 as the reference database. StrainPhlAn v4.0 was applied on SGB identified in the study models by considering those associated with a reference genome in NCBI with settings sample_with_n_markers=10, sample_with_n_markers_perc=10, sample_with_n_markers_after_filt = 20, sample_with_n_markers_after_filt_perc = 10, breadth_thres = 40.

Low-abundant species were removed using the nearZeroVar function of the caret R package, and only species detected in more than 30% of samples were selected for further analysis. Diversity metrics were computed using the vegan R package v2.5-6.1. The correlation network among identified features was generated using Cytoscape v3.10.3 considering the significant inter-omic Spearman correlations (adj. p <0.05) associated with a coefficient coherent among the three study cohorts.

***NMR analyses***

The list of metabolites quantified in serum using NMR (with corresponding ^1^H and ^13^C chemical shifts)

|  | **metabolite** | **^1^H chemical shift [ppm]** | **^13^C chemical shift [ppm]** |
| --- | --- | --- | --- |
| 1. | 3-Methyl-2-oxovalerate | 0.89 (t), **1.10** (d) | 13.3, 16.4 |
| 2. | 2-Hydroxybutyrate | **0.90** (t), 1.66 (m), 4.00 (m) | 11.4 |
| 3. | 2-Oxoisocaproate | **0.94** (d), 2.61 (d) | 24.5, 51.0 |
| 4. | Isoleucine | 0.94 (t), **1.01** (d), 1.27 (m), 1.47 (m), 3.68 (d) | 13.9, 17.4, 27.2, 38.6, 62.3 |
| 5. | Leucine | **0.96** (d), 0.97 (d), 1.71 (m), 1.72 (m), 3.73 (m) | 23.7, 24.8, 26.9, 42.6, 56.2 |
| 6. | Valine | 0.99 (d), **1.05** (d), 2.27 (m), 3.62 (d) | 19.4, 20.7, 31.8, 63.1 |
| 7. | 3-Hydroxyisobutyrate | **1.07** (d), 2.49 (m), 3.54 (m), 3.70 (m) | 16.8 |
| 8. | 2-Oxoisovalerate | **1.12** (d) | 19.1 |
| 9. | 2-Propanol | **1.18** (d) | n.d. |
| 10. | Ethanol | 1.19 (t), **3.65** (q) | n.d. |
| 11. | 3-Hydroxybutyrate | **1.20** (d), 2.31 (dd), 2.40 (dd) | 24.5, 68.4 |
| 12. | Lactate | 1.33 (d), **4.11** (q) | 22.9, 71.2 |
| 13. | Threonine | 1.33 (d), 3.59 (d), **4.25** (m) | 22.3, 63.2, 68.9 |
| 14. | Lysine | 1.45 (m), 1.52 (m), 1.73 (m), 1.91 (m), **3.04** (t) | 24.2, 29.1, 32.7, 41.8 |
| 15. | Alanine | **1.49** (d), 3.79 (q) | 18.9, 53.3 |
| 16. | Acetate | **1.92** (s) | 26.1 |
| 17. | Ornithine | 1.95 (m), **3.06** (t) | 30.3, 41.5 |
| 18. | Proline | 2.01 (dd), 2.08 (m), 2.36 (m), 3.43 (m), **4.14** (m) | 26.5, 31.8, 48.8, 63.9 |
| 19. | Glutamine | 2.14 (m), **2.46** (m) | 29.1, 33.6 |
| 20. | Acetone | **2.23** (s) | n.d. |
| 21. | Pyruvate | **2.38** (s) | n.d. |
| 22. | Citrate | 2.55 (d), **2.69** (d) | 48.5 |
| 23. | Dimethylamine | **2.72** (s) | 37.4 |
| 24. | Asparagine | **2.87** (dd), 2.95 (dd), 4.00 (m) | 37.5, 54.0 |
| 25. | Tyrosine | 3.06 (dd), 3.21 (dd), 3.95 (dd), **6.91** (m), 7.20 (m) | 38.3, 58.8, 118.6, 133.5 |
| 26. | Phenylalanine | 3.13 (dd), 3.29 (dd), 4.00 (m), 7.34 (m), 7.38 (m), **7.44** (m) | 39.1, 58.8, 130.4, 131.8, 132.2 |
| 27. | Glycine | **3.57** (s) | 44.2 |
| 28. | Glycerol | 3.56 (dd), **3.66** (dd) | 65.3 |
| 29. | Histidine | 3.99 (dd), **7.10** (s), 7.90 (s) | 57.4, 119.8, 138.9 |
| 30. | Tryptophan | 4.06 (dd), 7.21 (m), 7.29 (m), 7.33 (m), 7.55 (m), **7.74** (m) | 29.2, 57.8, 114.7, 121.2, 122.2, 124.8, 127.9 |
| 31. | Glucose | 4.66 (d), **5.25** (d)  3.26 (dd), 3.41 (m), 3,37 (m), 3.50 (dd), 3.55 (dd), 3.72 (m), 3.77 (dd), 3.84 (m), 3.90 (dd) | 94.8, 98.6  63.3, 63.5, 72.3, 74.2, 75.5, 76.9, 78.5, 78.7 |
| 32. | Mannose | 4.90 (d), **5.18** (d) | 96.4, 96.7 |
| 33. | Formate | **8.46** | n.d. |

The table lists of all detected signals of quantified metabolites; the signals used for metabolite quantification are in bold. Signal multiplicity is marked as follows: (s)-singlet, (d)-doublet, (t)-triplet, (dd)-doublet of doublets, (q)-quartet, (m)-multiplet; n.d. – signal not detected.

Representative ^1^H NMR spectrum of serum with quantified metabolites


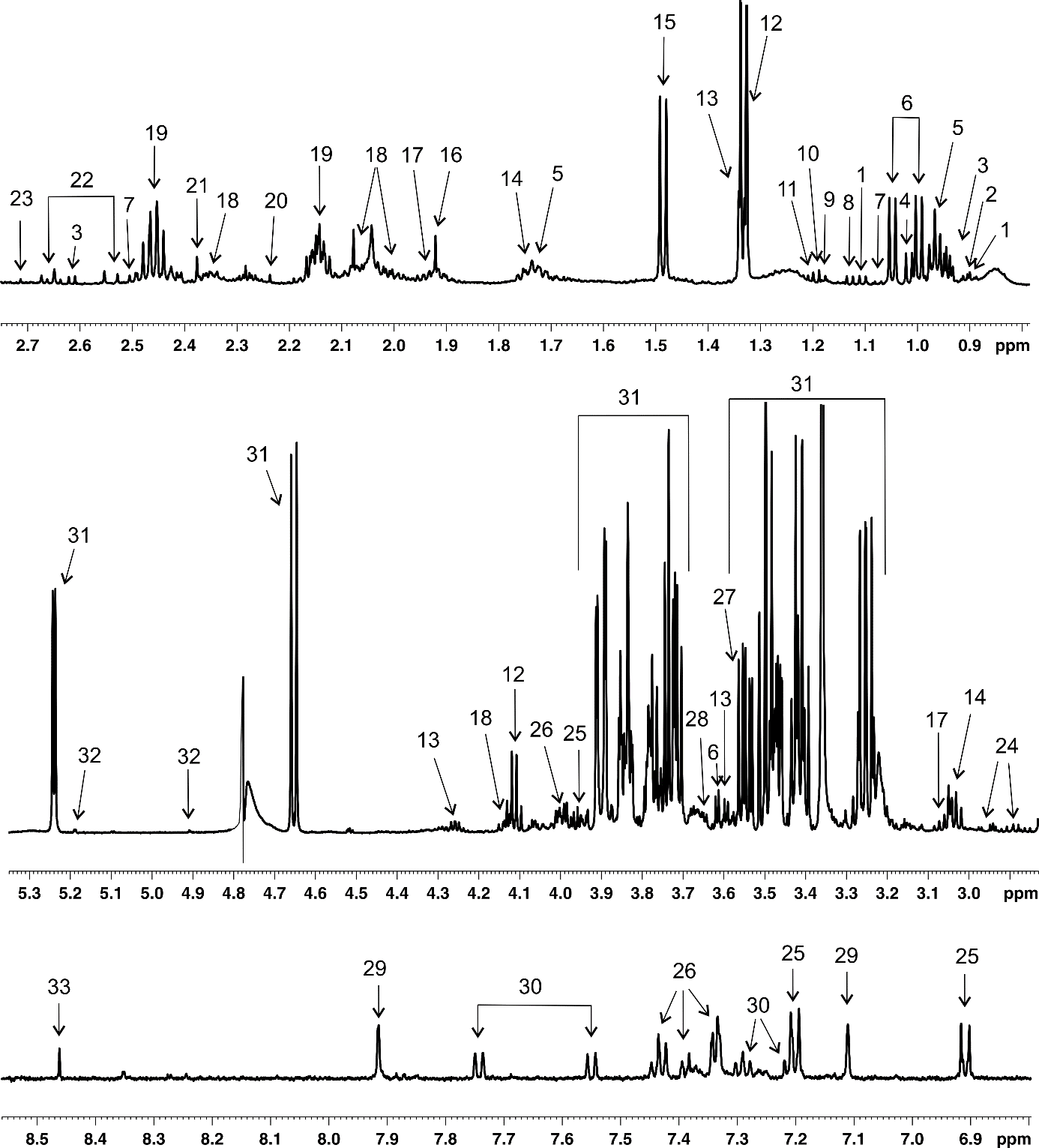


**Reference**

1. Thomas AM, Manghi P, Asnicar F, et al. Metagenomic analysis of colorectal cancer datasets identifies cross-cohort microbial diagnostic signatures and a link with choline degradation. *Nat Med* 2019;25(4):667-78. doi: 10.1038/s41591-019-0405-7 [published Online First: 20190401]

2. Blanco-Miguez A, Beghini F, Cumbo F, et al. Extending and improving metagenomic taxonomic profiling with uncharacterized species using MetaPhlAn 4. *Nat Biotechnol* 2023;41(11):1633-44. doi: 10.1038/s41587-023-01688-w [published Online First: 20230223]
