# Supplementary material for: A vegan diet signature from a multi-omics study on different European populations is related to favorable metabolic outcomes
Source: Gut Microbes. 2025 Dec 4;17(1):2593050. doi: 10.1080/19490976.2025.2593050 (PMC12688234; doi:10.1080/19490976.2025.2593050)
Supplement: Supplementary material [file KGMI_A_2593050_SM5051.docx]

**Supplementary Table 1.** Nutrient intake in vegans and omnivores, training cohort (*extension of the data reported in Table 1*).

|  | **VG_CZ** | **OM_CZ** | **VG_IT** | **OM_IT** | **Dunn’s multiple comparison test** | | | |
| --- | --- | --- | --- | --- | --- | --- | --- | --- |
|  |  |  |  |  | VG_CZ vs OM_CZ | VG_IT vs OM_IT | OM_CZ vs OM_IT | VG_CZ vs  VG_IT |
| **Macronutrient intake** | | | | | | | | |
| Total energy  [kcal. day^-1^] | 2514 (793) | 2260 (900) | 1523 (771) | 2025 (1203) | 0.139 | 0.017 | 0.245 | <0.001 |
| Total lipids  [g. day^-1^] | 85.8  (37.5) | 95.0  (53.6) | 62.4  (25.0) | 72.6  (52.3) | 0.146 | <0.001 | 0.021 | <0.001 |
| Proteins  [g. day^-1^] | 80.6 (35.6) | 88.0 (40.6) | 62.4 (23.2) | 78.9 (44.9) | 0.628 | 0.058 | 0.526 | 0.001 |
| Carbohydrates  [g. day^-1^] | 297  (106) | 236 (122) | 209  (86) | 245 (143) | 0.001 | 0.456 | 0.895 | <0.001 |
| Sugars  [g. day^-1^] | 94.6 (58.2) | 69.1 (25.2) | 101.4 (62.6) | 98.1 (79.9) | 0.008 | 0.813 | 0.008 | 0.813 |
| Dietary fiber  [g. day^-1^] | 43.0 (22.1) | 17.3 (7.8) | 28.8 (14.8) | 18.2 (12.7) | <0.001 | <0.001 | 0.610 | <0.001 |
| Cholesterol  [mg. day^-1^] | 0.2  (3.2) | 353.0 (295.7) | 55.4 (60.4) | 321.0 (184.3) | <0.001 | <0.001 | <0.001 | <0.001 |
| SFA  [g. day^-1^] | 20.5 (15.2) | 35.8 (20.2) | 14.8 (7.3) | 28.1 (19.1) | <0.001 | 0.680 | 0.680 | <0.001 |
| MUFA  [g. day^-1^] | 23.9 (16.2) | 24.3 (16.3) | 35.6  (16.1) | 38.0  (28.5) | 0.980 | 0.390 | <0.001 | <0.001 |
| PUFA  [g. day^-1^] | 18.6 (13.9) | 9.7  (6.4) | 8.4 (4.4) | 7.2  (5.9) | <0.001 | <0.001 | <0.001 | <0.001 |

Data are given as median (interquartile range). The overall difference among groups was evaluated using Kruskal-Wallis test followed by Dunn´s multiple comparison test. MUFA, mono-unsaturated fatty acids; N/A, not applicable; PUFA, poly-unsaturated fatty acids; SFA, saturated fatty acids.
